# Development of a novel Haemabiome tool for the high-throughput analysis of haemopathogen species co-infections in African livestock

**DOI:** 10.3389/fvets.2024.1491828

**Published:** 2024-12-20

**Authors:** Erhan Yalcindag, Deepali Vasoya, Johanneke D. Hemmink, Benedict Karani, Luis Enrique Hernandez Castro, Rebecca Callaby, Stella Mazeri, Edith Paxton, Timothy K. Connelley, Phil Toye, Liam J. Morrison, Barend Mark de C. Bronsvoort

**Affiliations:** ^1^Centre for Tropical Livestock Genetics and Health (CTLGH), Roslin Institute, University of Edinburgh, Edinburgh, United Kingdom; ^2^The Roslin Institute and Royal (Dick) School of Veterinary Studies, University of Edinburgh, Edinburgh, United Kingdom; ^3^Centre for Tropical Livestock Genetics and Health (CTLGH), ILRI Kenya, Nairobi, Kenya; ^4^The International Livestock Research Institute, Nairobi, Kenya

**Keywords:** high-throughput amplicon sequencing, molecular diagnosis, haemopathogens, cattle, Africa

## Abstract

One of the principal limitations on livestock productivity in sub-Saharan Africa is the constraining effect of infectious diseases, including tick-borne blood pathogens. Currently, diagnostic markers for these pathogens are species or genus specific, making it challenging to implement high-throughput screening methods. The aim of this study was to develop and validate a novel high-throughput diagnostic tool capable of detecting a range of important haemopathogens in livestock. To achieve this, we developed a high-throughput diagnostic tool that can detect all species of *Anaplasma*, *Ehrlichia, Theileria* and *Babesia* present in a sample. The approach involves targeting the 16S/18S rDNA region by PCR and subjecting amplicons to deep sequencing, which allows for the identification of species present in a sample, and the exploration of haemopathogen communities. To validate the accuracy of this Next Generation Sequencing method, we compared the amplicon sequencing results with species-specific PCR and reverse line blot (RLB) test data of both control and field samples. The Haemabiome tool demonstrated the successful resolution of positive and negative samples, and highlighted the power of this diagnostic tool in identifying multiplicity of infections. The Haemabiome tool can therefore generate valuable insights regarding the understanding of the true diversity of species composition and the distribution of pathogen communities in field samples.

## 1 Introduction

Infectious diseases cause major costs and constraints to livestock production. It is estimated that over 600 million people globally depend on livestock, and represent up to 70% of the population in the most marginal areas (1, 2). In low and middle income countries (LMICs), particularly in sub-Saharan Africa (SSA), cattle are infected by a range of economically important and endemic diseases, each with distinct epidemiological properties (3). Among those affecting cattle in SSA, tick-borne pathogens collectively represent some of the most impactful including multiple piroplasma species such as *Theileria* and *Babesia*, and Rickettsia such as *Anaplasma* and *Ehrlichia*. These organisms are termed ‘haemopathogens’ as they are predominantly bloodstream based in the mammalian host (4–7). Haemopathogens are a particularly important group of pathogens in Africa and contribute to high levels of mortality in cattle (8, 9).

Bovine piroplasmosis is caused by the tick-borne *Babesia* and *Theileria* spp. which are arguably some of most economically important livestock parasites (10, 11). Within these genera, some species are highly pathogenic, such as *Theileria parva*, the main agent of East Coast fever and corridor disease, which kills over 1 million cattle each year in SSA (11–13). Other *Theileria* species circulating in Africa include *T. mutans*, *T. taurotragi*, *T. sergenti/buffeli/orientalis* (referred to as the *T. orientalis* complex) and *T. velifera* (14), all of which are considered to be either less pathogenic or not pathogenic in cattle, and are reported to cause benign, moderate or asymptomatic theileriosis (15). In Africa, bovine babesiosis is caused by *Babesia bovis* and *B. bigemina* (16). While *B. bigemina* is more prevalent, *B. bovis* infection results in a greater disease burden because of the neurological symptoms associated with infection (17). Anaplasmosis and Ehrlichiosis are also highly important tick-borne ruminant diseases in SSA. Heartwater, caused by *Ehrlichia ruminantium* (18, 19), and infections with *Anaplasma centrale*, *A. phagocytophilum*, *A. bovis* and *A. marginale,* are widespread (20, 21). The latter is known to be pathogenic to domestic ruminants, particularly to high producing dairy cattle (22).

Historically, understanding of these pathogens has progressed through investigation and study of one pathogen at a time using a range of diagnostic approaches to detect the specific pathogens, including microscopy (8), antibody-based techniques such as ELISA (23, 24), and several PCR methods including conventional PCR, reverse line blot (RLB)-PCR, quantitative qPCR and multiplex PCR (25–29). However, it is clear that co-infections are both common and important, and that pathogen combinations can work together (synergistically) or against each other (antagonistically) (30, 31). Additionally, many pathogen infections cause immunosuppression, such that infection with one pathogen increases the chance that a host will be infected with another. In SSA, co-infection, including with species of the same genus or group of genera, are common in livestock (8, 32) and relevant at both the level of small holder farmers and commercial ranching operations.

If we are to understand the complex interactions that occur in the multiple-pathogen disease ecosystem in sub-Saharan livestock—which potentially influence the prevalence of other pathogens, cattle productivity, and the effectiveness of interventions—it is essential to use tools that can identify multiple pathogens. These tools will be crucial for generating the requisite data at scale. Next Generation Sequencing (NGS) technologies represent powerful approaches to enable the investigation of the population genetics, ecology and dynamics of pathogen communities across a range of taxonomic scales (33). NGS targeting one region of DNA provides millions of sequences with low error rates, making it feasible to investigate species diversity and prevalence in large populations (34–36). One approach involves PCR amplification using custom-designed oligonucleotide primers targeting the 16S/18S ribosomal DNA of the pathogen, a suitable target due to the highly conserved sequences flanking this region. The significant species-specific variation within this region then allows for the discrimination between species (37, 38), and potentially also facilitates the distinction between strains or subtypes (depending on coverage) (36, 39). This approach has previously been applied to *Theileria* and *Babesia* (32, 36, 40–45), demonstrating the robustness of the NGS method. The amplified PCR products can then be subjected to multiplex amplicon sequencing that enables high throughput data generation from hundreds or thousands of samples, with a custom-designed bioinformatics pipeline facilitating downstream deconvolution of data per sample and allocation of sequenced reads to pathogen species. Using multiplexed barcoded primer combinations, up to 384 samples can be processed simultaneously on a single Illumina MiSeq flow cell, significantly reducing costs.

The aim of this study was to develop and validate a novel high-throughput diagnostic tool capable of detecting a range of important haemopathogens in livestock, which would remove the need to individually screen for one pathogen at a time. Compared to previous studies, we have now also included pathogens from the *Ehrlichia* and *Anaplasma* genera. We hereafter refer to the platform as the Haemabiome tool, and see its potential utility for the in-depth elucidating of co-infections, allowing simultaneous detection of many of the most important vector-borne livestock pathogens (the tool specifically targets any species in the *Theileria*, *Babesia*, *Erhlichia* and *Anaplasma* genera) with high confidence. This sequencing approach will also allow the detection of both pathogenic and low/non-pathogenic subspecies within the genera, and opens up the study of co-infections in ways previously not possible (32, 36). It also has future application potential to clinical diagnostics in the field.

## 2 Materials and methods

### 2.1 Positive control samples

DNA from laboratory cultured strains of pathogens or verified single-pathogen infections were used as single-species control samples to evaluate the specificity of primer pairs, and this panel included *Anaplasma phagocytophilum*, *Ehrlichia ruminantium*, *Babesia bigemina*, *Babesia bovis*, *Theileria annulata*, *Theileria mutans*, *Theileria parva* and *Theileria taurotragi* (for more details see Table 1).

**Table 1:** Samples used as positive controls to validate the deep amplicon sequencing approach for the accurate detection of species composition.

Species	Strain	Type of sample	Reference laboratory
*Anaplasma marginale*	D641	NA	Washington State University
*Anaplasma phagocytophilum*	NA	Cultured in tick cells	Tick cell biobank/University of Liverpool
*Ehrlichia ruminantium*	Gardel	Feral Goat strain	Tick cell biobank/University of Liverpool
*Babesia bigemina*	NA	Stabilate 4635	ILRI (International Livestock Research Institute)
*Babesia bovis*	K	Cultured in tick cells	Roslin Institute/University of Edinburgh
*Theileria annulata*	Ankara	Cell line	Roslin Institute/University of Edinburgh
*Theileria mutans*	Zanzibar	Stabilate 3778	ILRI (International Livestock Research Institute)
*Theileria parva*	Muguga	Cell line	Roslin Institute/University of Edinburgh
*Theileria taurotragi*	NA	Field sample	Roslin Institute/University of Edinburgh

NA, Not applicable.

### 2.2 Field samples

Samples originally collected from the Infectious Diseases of East African Livestock (IDEAL) calf cohort study conducted in Kenya from 2007 to 2009 were used (8). In this cohort study, calves were followed from birth up to 1 year old. These calves did not receive any preventive vaccines or treatments during this period in order to measure true cumulative pathogen exposure. For the purposes of tool validation, we examined samples from a subset of 31 calves from the IDEAL study in three categories: (1) nine animals with clinical episodes that survived, (2) nine animals without clinical episodes that survived and (3) 13 animals not categorised. In addition, a subset of eight of these IDEAL samples, underwent sequencing across different runs to assess reproducibility of results. These particular samples will be henceforth referred to as “repeated samples.” A total of 279 Kenyan cattle blood samples from the above-mentioned animals were tested in this study (Supplementary Table S1). These subsets were selected as they can provide insight into tool validation with respect to calves that are more likely to be infected (‘clinical disease’ cohort), as well as the degree of infection/co-infection occurring in calves that did not undergo clinical episodes (‘no clinical episodes’), and animals that were previously diagnosed to be infected with African trypanosomes, to assess diversity in animals infected with a pathogen known to cause immunosuppression (46) and therefore potentially predispose to multiplicity of infection (‘trypanosomes’).

### 2.3 DNA extraction

DNA was extracted from 100 μL of blood originally collected from the calves in the IDEAL study and stored in the ILRI liquid nitrogen biobank, using the Qiagen DNA blood and tissue kit according to the manufacturer’s recommendation, with the modification of incubating blood with lysis buffer for 30 min to allow full lysis of the cattle blood. A control extraction was also carried out using distilled water as a substrate, and this was subsequently used as a negative control to test for any cross-contamination that may have occurred during DNA extraction.

### 2.4 Primer design and validation

For the amplification of parasites from the *Theileria* and *Babesia* genera, the V4 hypervariable fragment (378–424 bp) of the 18S rRNA gene was targeted. Primers were adapted from the primers previously described by Bishop et al. (47); RLB-F (28) and RLB-R2 (48) (Table 2).

**Table 2:** Primer sequences for first round PCR targeting genus level amplification.

Genus	Target gene	Primer name	Sequences (5′–3′) with adapter primers	Amplicon size (bp)	Reference
*Anaplasma*/*Ehrlichia*	16 s rDNA	AE-F4	*TCGTCGGCAGCGTCAGATGTGTATAAGAGACAG*CGCAAGACTAAAACTCAAAGGAATTGACG	~210	This study
		AE-R3	*GTCTCGTGGGCTCGGAGATGTGTATAAGAGACAG*CTCGTTGCGGGACTTAACCCAAC		
*Theileria*/*Babesia*	18 s rDNA	RLB-F	*TCGTCGGCAGCGTCAGATGTGTATAAGAGACAG*GAGGTAGTGACAAGRAATAMCAATA	~430	(28)
		RLB-R2	*GTCTCGTGGGCTCGGAGATGTGTATAAGAGACAG*CTAAGAATTTCACCTCTGACAGT		(48)

The adapter sequences are shown in italics. The direction is indicated as either “F” for forward or “R” for reverse.

For *Anaplasma* sp. and *Ehrlichia* sp., primers were designed in the homologous regions of the variable regions of 16S rDNA based on the 23 *Anaplasma* sp. and *Ehrlichia* sp. sequences obtained from NCBI database (Supplementary Table S2) and their specificity was confirmed by blasting the primer sequence against the NCBI database. Different combinations of primers, predicted to give an amplicon size of between 100 and 600 bp, were tested using a panel of control samples with known pathogen content, to validate their ability to amplify samples with *Anaplasma* sp. and *Ehrlichia* sp., but not other haemopathogens. Based on the validation results, the AE-F4 and AE-R3 primer pair was selected (Table 2).

### 2.5 Generation of 16S/18S rDNA PCR amplicons for sequencing

In order to generate amplicons with unique identifiers ready for sequencing, two rounds of PCR were performed. The first-round targeted amplification of the genus specific sequences of the 16S/18S rDNA locus using primers which included adapter sequences to facilitate the second round of PCR. The second round of amplification was performed to anneal the Nextera II multiplex identifier tags (MID) and sequencing primers (Supplementary Table S3).

The two sets of genus specific PCRs; *Theileria/Babesia* (referred to hereafter as “ThBa”) and *Anaplasma*/*Ehrlichia* (referred to hereafter as “AnEh”) were conducted in separate 20 μL reactions, each containing 4 μL Phusion high-fidelity PCR master mix (New England Biolabs), 0.2 μL HF Phusion Taq Polymerase, 10 mM dNTPs, 0.6 μL DMSO, 10 μM of each forward and reverse primer, and 2 μL of template DNA. Cycling conditions were as follows: *Theileria/Babesia*: 98°C (30 s), 25 cycles of 98°C (10 s), 60°C (30 s) and 72°C (30 s), with 10 min at 72°C; *Anaplasma/Ehrlichia*: 98°C (30 s), 25 cycles of 98°C (10 s), 64°C (20 s), 72°C (20 s) with 10 min at 72°C. For initial assessment of amplification, some of the amplified products were subjected to electrophoresis in a 1.5% agarose gel in TAE buffer and visualised under ultraviolet light. PCR products were then purified using AMPure XP magnetic beads (Beckman Coulter) according to manufacturer’s instructions.

The second round PCR was performed by using combinations of 16 forward (N502 to N511, N513 to N522) and 12 reverse barcoded primers (N701 to N715) (Supplementary Table S3) so that each individual sample in a pool was assigned a unique barcode combination. PCRs were performed using the same reaction mix as described for the first round PCR, except for the addition of 10 μM barcoded forward and reverse primers, and 1 μL of first round PCR product as a template. Cycling conditions were as follows: *Theileria/Babesia*: 98°C (30 s), 10 cycles of 98°C (10 s), 64°C (30 s), 72°C (30 s), and 10 min at 72°C. *Anaplasma/Ehrlichia*: 98°C (30 s), 10 cycles of 98°C (10 s), 64°C (20 s), 72°C (20 s), and 5 min at 72°C. 10 μL of each sample was pooled to create a master sequencing library pool. Then, 100 μL from each pool were loaded onto a 1.5% agarose gel. The products were then purified using by Qiagen QIAquick gel extraction kit (Qiagen) and subsequently with AMPure XP magnetic beads (1X), following the manufacturer’s instructions (Beckman Coulter). Each pool was then quantified using Qubit (Invitrogen) according to the manufacturer’s instructions. Finally, 70 μL of purified pool, at a concentration of 50 nM for AnEh and 22 nM for ThBa, was submitted for sequencing on an Illumina MiSeq platform using a 500-cycle paired-end reagent kit (MiSeq Reagent Kits v2, 2 × 250 bp paired-end reads) with 10% PhiX Control v3 (Supplementary Figure S2). Each of the pools were sequenced in a separate lane.

The first round PCR product of all samples was assessed for positivity on an agarose gel. Based on the results observed on the gel, we proceeded to create two different libraries for each infection. The first library consisted of samples that showed the expected positive band on the gel, and included selected positive and negative control samples. The second library comprised samples that did not show any visible bands by gel electrophoresis in the first PCR. These samples were pooled separately, also including selected positive and negative samples, and eight repeated samples from the first library to assess the haemobiome tool’s reproducibility.

### 2.6 Species-specific PCR validation

In order to compare the haemabiome results with data derived from well characterised species-specific assays, specific primers targeting *T. parva* sporozoite microneme-rhoptry surface antigen (p104) and the *A. bovis* 16S rRNA gene were amplified by a two-step semi-nested PCR using forward and reverse primers as previously described (49, 50). For *T. parva* p104, the first round PCR was performed using 20 μL reactions containing 4 μL Phusion high-fidelity PCR master mix (New England Biolabs), 0.2 μL HF Phusion Taq Polymerase, 10 mM dNTPs, 10 μM of each forward and reverse primer, 11.8 μL ddH20 and 1 μL of template. p104 first round PCR cycling conditions were 98°C (30 s), [98°C (10 s), 62°C (30 s), 72°C (20 s min)] for 30 cycles, and 2 min at 72°C. For second round PCR, 1 μL of first PCR product as template with the same conditions, except the annealing temperature which was 66°C.

For the *A. bovis* 16S gene, PCR was performed using 20 μL reactions containing 10 μL Q5 High-Fidelity Reaction Buffer (New England Biolabs), 10 μM of each forward and reverse primer, 7 μL ddH20 and 1 μL of template. The first round PCR cycling conditions were 98°C (1 min), [98°C (1 min), 56°C (1 min), 72°C (1:30 min)] for 35 cycles and 5 min at 72°C.

For the second round PCR, 1 μL of first PCR product was used as template with PCR conditions of 98°C (30 s), [98°C (10 s), 62°C (30 s), 72°C (30 s)] for 30 cycles and 2 min at 72°C.

### 2.7 Bioinformatic analysis

**Figure 1:**
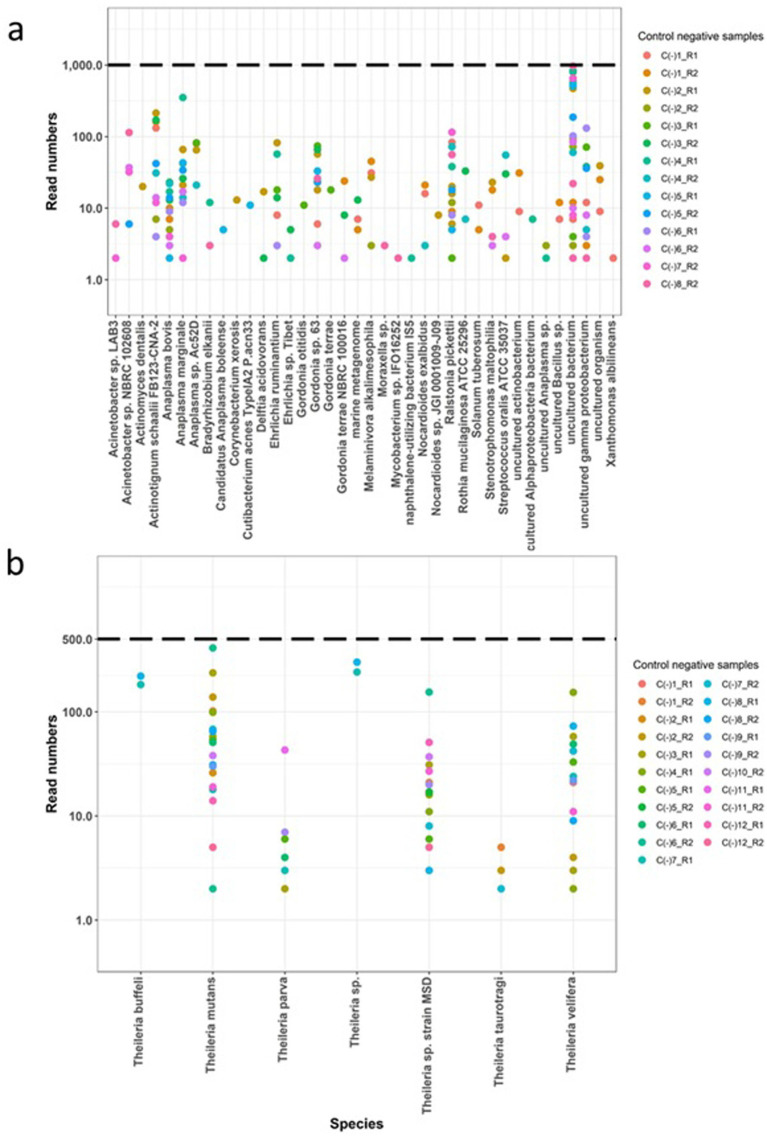
Assessment of the Haemabiome tool on the negative control samples. Negative controls from each run (different coloured dots) were filtered for number of reads (y-axis) and sequence alignment (x-axis) for each genus. Obtained filtered read numbers presented in y-axis based on log 10. Dotted lines present threshold. C (−): Negative control samples. R: Tested run ID. **(A)** Tested *Anaplasma*/*Ehrlichia* sp. negative control samples; **(B)** tested *Theileria*/*Babesia* sp. negative control samples.

**Figure 2:**
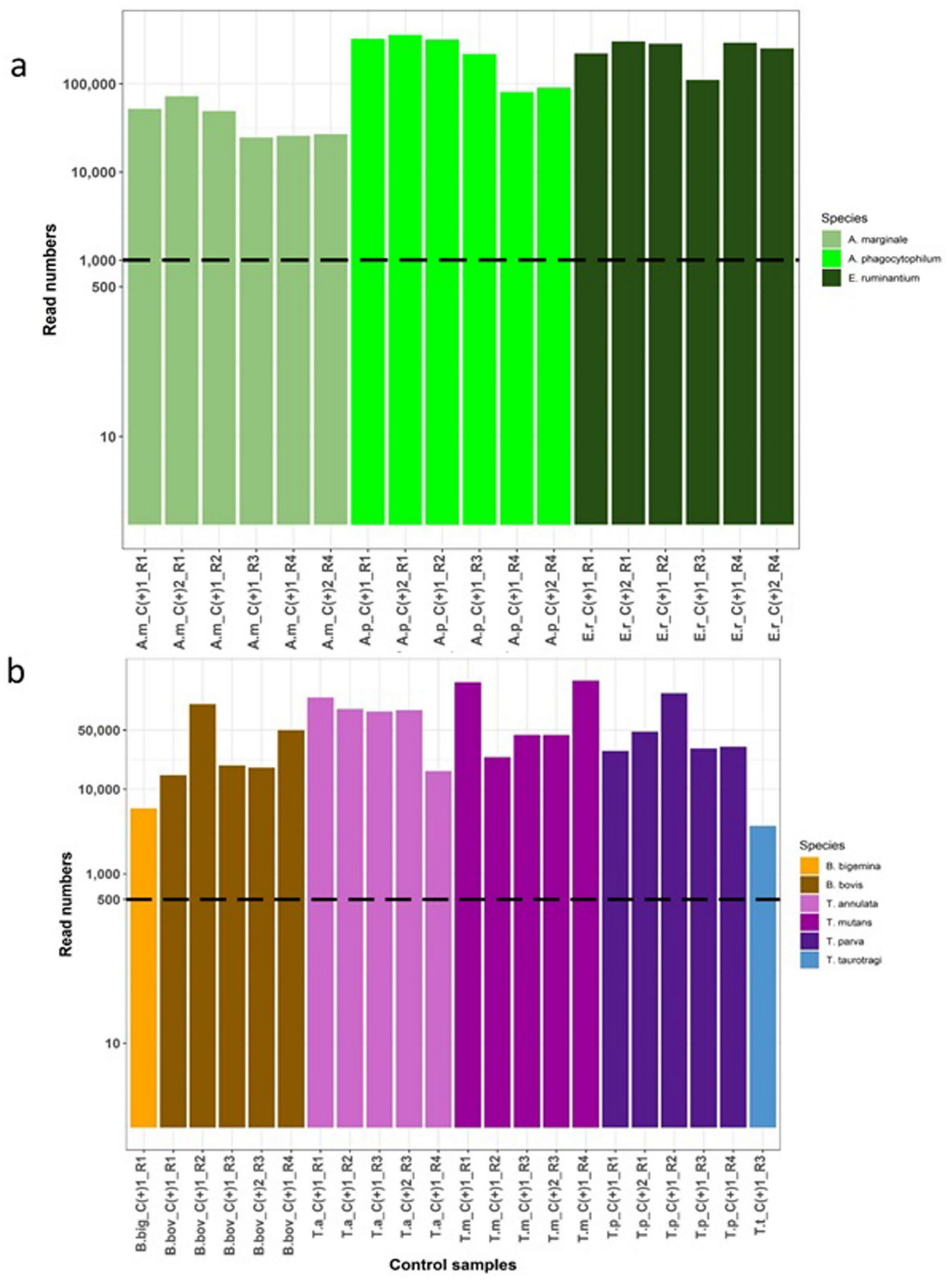
Assessment of the Haemabiome tool on species-specific positive control samples. For each genus, a number of positive control samples on different runs were tested and are highlighted in different colours by genus. The filtered read numbers are presented in the y-axis based on a log_10_ scale. Individual samples are labelled on the x-axis where the coding is as follows: R: Test run ID. C+: Control |positive sample. **(A)** Validation of *Anaplasma*/*Ehrlichia* sp. (AnEh). A.m: *A. marginale*, A.p: *A. phagocytophilum*, E.r: *E. ruminantium*. **(B)** Validation of *Theileria*/*Babesia* sp. (Thba), B. big: *B. bigemina,* B. bov: *B. bovis*, T. a: *T. annulata*, T.m: *T. mutans*, T.p: *T. parva*, T.t: *T. taurotragi*.

Amplicon libraries were submitted to Edinburgh Genomics Core facility to generate 250 bp paired end sequencing reads on Illumina MiSeq v2 platform. The raw sequencing data has been submitted to the European Nucleotide Archive and is available under the project accession number PRJEB79313. Sequencing reads were deconvoluted into data from individual samples based on the barcoding combinations. To ensure data quality, the raw sequences were evaluated using Fastqc and poor quality reads with a Phred score below 28 were removed using Sickle (51). The resulting high quality paired end reads were merged using Flash (52) to produce extended amplicon sequences. These merged sequences were then separated based on the presence of primers used for AnEh and ThBa. Within data from each sample, sequences that were 100% identical were grouped into clusters. However, clusters were excluded from further analysis if they met the following criteria: (1) they represented a single copy sequence read, (2) they were predicted to be formed as a PCR chimera from two other more frequent cluster sequences within the same sample, or (3) if they differed by only one nucleotide from a cluster sequence present in the same sample with higher read counts and a fold change of 3, as these clusters may have arisen from sequencing or PCR errors and could not confidently be defined as distinct. The remaining clusters were compared to the SILVA database v138.1 (18S/16S rRNA) using BLAST (53). Following the data from the control samples, specific criteria were established to define positive infections in each sample. These measures comprised the following rules: (a) establishing read frequency thresholds of 1,000 for AnEh and 500 for ThBa mapped clusters (Figures 1A,B, 2A,B), (b) setting percentage identity thresholds of 99 and 97% for AnEh and ThBa, respectively, and (c) ensuring sequence lengths were within the expected ranges of 160–162 bp for *Anaplasma*/*Ehrlichia* and 330–378 bp for *Theileria*/*Babesia*, respectively (Supplementary Figure S3).

### 2.8 Sequence alignment, sequences and phylogenetic analyses

The obtained partial 16S/18S sequences were aligned with reference sequences from Genbank and existing literature listed in Supplementary Tables S2, S4, using Clustal W with Bioedit software version 7.2.5 (54). Phylogenetic trees were constructed using trimmed partial 16/18S gene sequences from both the IDEAL sample data and aforementioned reference sequences.

To determine the best available evolutionary models for Bayesian inference (BI), we used JModelTest version 0.1.1.40 (55) and selected models based on Akaike Information Criterion (AIC). The Bayesian analysis was performed using MrBayes 3.2.6_168 (56) through the phylogeny web service.^1^ The Bayesian analysis processed two simultaneous independent runs with four chains each proceeding for 1,000,000 generations. Trees were sampled every 250 generations. The first 25% of iterations were excluded as burn-in. Resulting trees were combined to create a majority-rule consensus tree, from which posterior probabilities were obtained. A *Paracoccus* sp. (GenBank accession KP003988) and the *Hemalivia stellate* (GenBank accession KP881349) sequences were used as outgroups for constructing the AnEh and ThBa trees, respectively, following the study by Chiuya et al. (57).

### 2.9 Statistical analysis

Species-specific nested PCR results were taken as the “gold standard” and compared with the Miseq results. Using the constructed gold standard, sensitivity and specificity were estimated using the “medcalc” software version 19.2.6 (58). Agreement between the different diagnostic tests (species-specific nested PCR and Miseq) assessing the presence of *A. bovis* and *T. parva* was calculated. A kappa measure of agreement test was performed to compare the performance of the two tests; *κ*-value < 0 indicates no agreement beyond chance. A *κ*-value between 0.41 and 0.60 indicates a moderate level of agreement while a *κ*-value between 0.81 and 0.99 indicates almost perfect agreement (59). Other statistical analyses were carried out using R version 3.5.1.

## 3 Results

### 3.1 Comparison of the Haemabiome tool with known controls, replicates and species-specific primers

To validate the specificity of the Haemabiome tool, we performed the two PCRs using the primer sets described in Table 2 on the set of negative control samples (water negative controls processed using the DNA extraction protocol). We used these results to set the selected filtering thresholds for each pathogen genus (AnEh <1,000 reads and ThBa <500 reads, respectively). Although there are small numbers of reads present in the negative control samples, these are generally well below thresholds and can be confidently assigned as ‘false positives.’

Moreover, for the AnEh primers several bacterial genera were present in some negative controls, which were previously reported as contaminants in DNA extraction kits, PCR and other laboratory reagents. This included *Acinetobacter*, *Actinomyces*, *Bradyrhizobium*, *Corynebacterium*, *Cutibacterium*, *Delftia*, *Moraxella*, *Ralstonia*, *Stenotrophomonas*, *Streptococcus*, and *Xanthomonas* (60, 61). Although detected with very few reads (<100), other bacterial genera such as *Gordonia* and *Nocardioides* could also be considered potential contaminants of the 16S rDNA NGS process (Figure 1A) for the AnEh primers. However, some negative control samples from the AnEh PCRs did generate read numbers that approached the threshold of 1,000 reads (Figure 1A). When checked, the sequences in these reads aligned with “uncultured bacterium,” indicating clear false positives. These data provided confidence that the tool was not generating false positives above the defined threshold (Figures 1A,B).

Sequencing replicates were carried out using the species-specific positive control DNA (Table 1) to validate the consistency, accuracy and reliability of deep amplicon sequencing. Read numbers for each replicate set are shown in Figure 2. The results showed that the Haemabiome tool successfully amplified and detected all positive control samples, albeit with varying read numbers between different sequencing runs (Figure 2).

**Figure 3:**
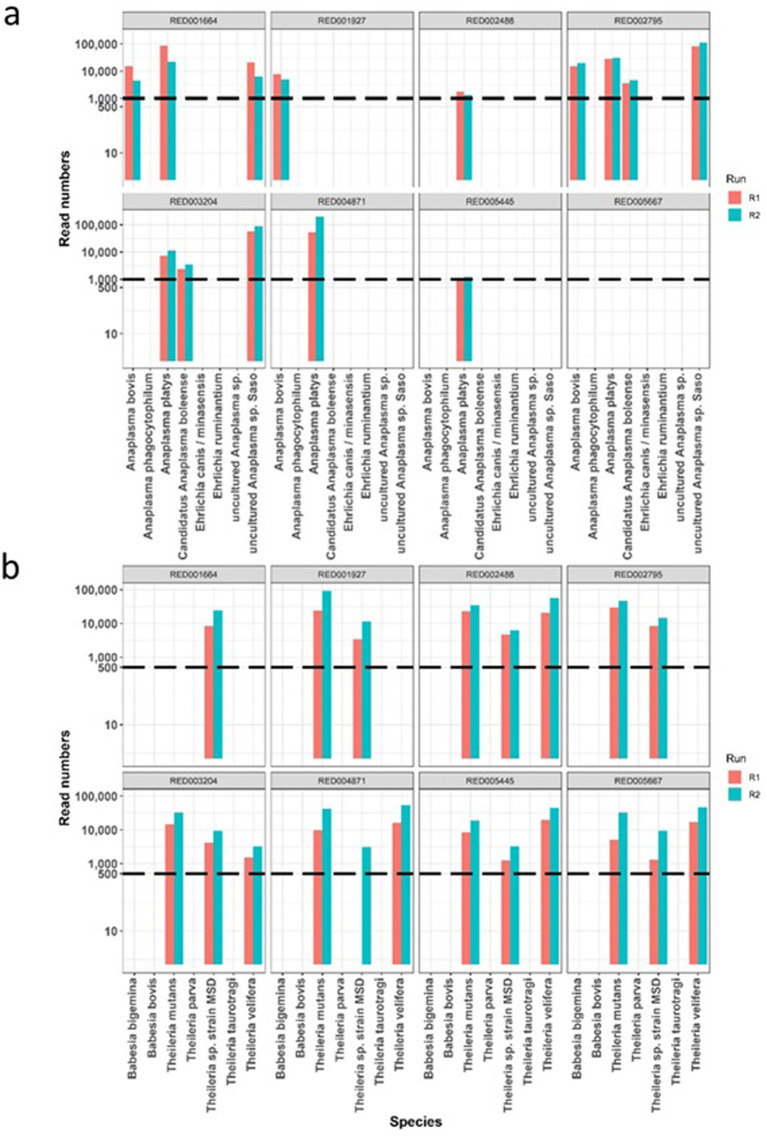
The comparison of read counts repeated across two different runs for eight individual sample (one panel per sample), with pathogen species names on the x-axis. Dotted lines mark the thresholds identified from Figure 1. **(A)** Read numbers for *Anaplasma*/*Ehrlichia* species. **(B)** Read numbers for *Theileria*/*Babesia* species.

To assess reproducibility, eight field samples were subjected to repeat testing in different sequencing runs for both AnEh and ThBa primer sets (Figure 3). In each distinct run, the same pathogen species were consistently detected within each tested sample, and the number of reads linked to each species showed similar relative patterns between repeats. This consistent detection and comparable pattern of read counts indicated that the Haemabiome tool successfully identified the presence of the same pathogen species in both runs in similar proportions, confirming reliability and accuracy of the approach for each pathogen genus across independent sequencing runs.

Finally, as part of the validation we utilised known species-specific primers for the selected individual pathogens, to confirm the Miseq data generated using the Haemabiome tool and the IDEAL samples. For the amplification of *A. bovis* a nested PCR targeting the species-specific16S gene was used (50). For *T. parva* we used a species-specific nested PCR targeting the *T. parva* p104 gene (49).

A total of 279 samples from the 31 animals were screened. Of these, 179 (64.8%) were positive for *A. bovis* by species-specific nested PCR, compared to 138 (49.4%) samples testing positive using the Haemabiome tool (Table 3). The calculated *κ*-value, which measures the agreement between the two tests, for *A. bovis* detection, indicated a substantial level of agreement (*κ* = 0.65) (Table 4).

**Table 3:** Comparison of conventional nested PCR and Miseq (Haemabiome tool) results for *Anaplasma bovis* and *Theileria parva* for the 279 samples from 31 calves from the IDEAL study.

Genus	Species	Total Miseq positive sample	Total Miseq negative samples	Overall proportion (%95)	Nested PCR positive samples	Nested PCR negative samples	Overall proportion (%95)
AnEh	*A. bovis*	138	141	49.4 (43.6–55.3)	179	100	64.8 (58.9–70.4)
ThBa	*T. parva*	23	256	8.2 (5.5–12.2)	83	196	29.7 (24.5–35.5)

**Table 4:** Comparison of species-specific PCR and Haemabiome tool for *A. bovis* and *T. parva* detection.

		Gold standard (16S/p104)		Sensitivity % (95% CI)	Specificity % (95% CI)	Accuracy % (95% CI)	Kappa value
		Positive	Negative				
Screening test (AnEh)							
Miseq (*A. bovis*)	Positive	134	4	77.9 (67.8–81.3)	96 (90.1–98.9)	82.4 (77.4–86.7)	0.65
	Negative	45	96				
Screening test (ThBa)							
Miseq (*T. parva*)	Positive	19	4	22.9 (14.9–33.42)	98 (94.9–99.4)	81.7 (66.3–92.1)	0.26
	Negative	64	192				

A total of 83 (29.7%) samples were positive by the p104 nested PCR for *T. parva*, while only 23 (8.2%) samples were positive on the Miseq assay (Table 3). The calculated kappa value of 0.26 indicated only slight or weak agreement between the PCR and Miseq tests for the presence of *T. parva* infections (Table 4).

Using the species-specific PCR results as the gold standard we estimated the sensitivity and specificity of the Haemabiome tool (Table 4). The sensitivity and specificity of the Haemabiome tool were estimated as 77.9 and 96%, respectively for *A. bovis*, resulting in an overall accuracy of 82.4%. On the other hand, the Haemabiome tool had an estimated sensitivity of 22.9% and specificity of 98% for *T. parva*, resulting in an accuracy of 81.7%.

This suggests that the Haemabiome tool may be significantly less sensitive than the nested PCRs, which is to be expected given the high sensitivity of the latter tests.

**Figure 4:**
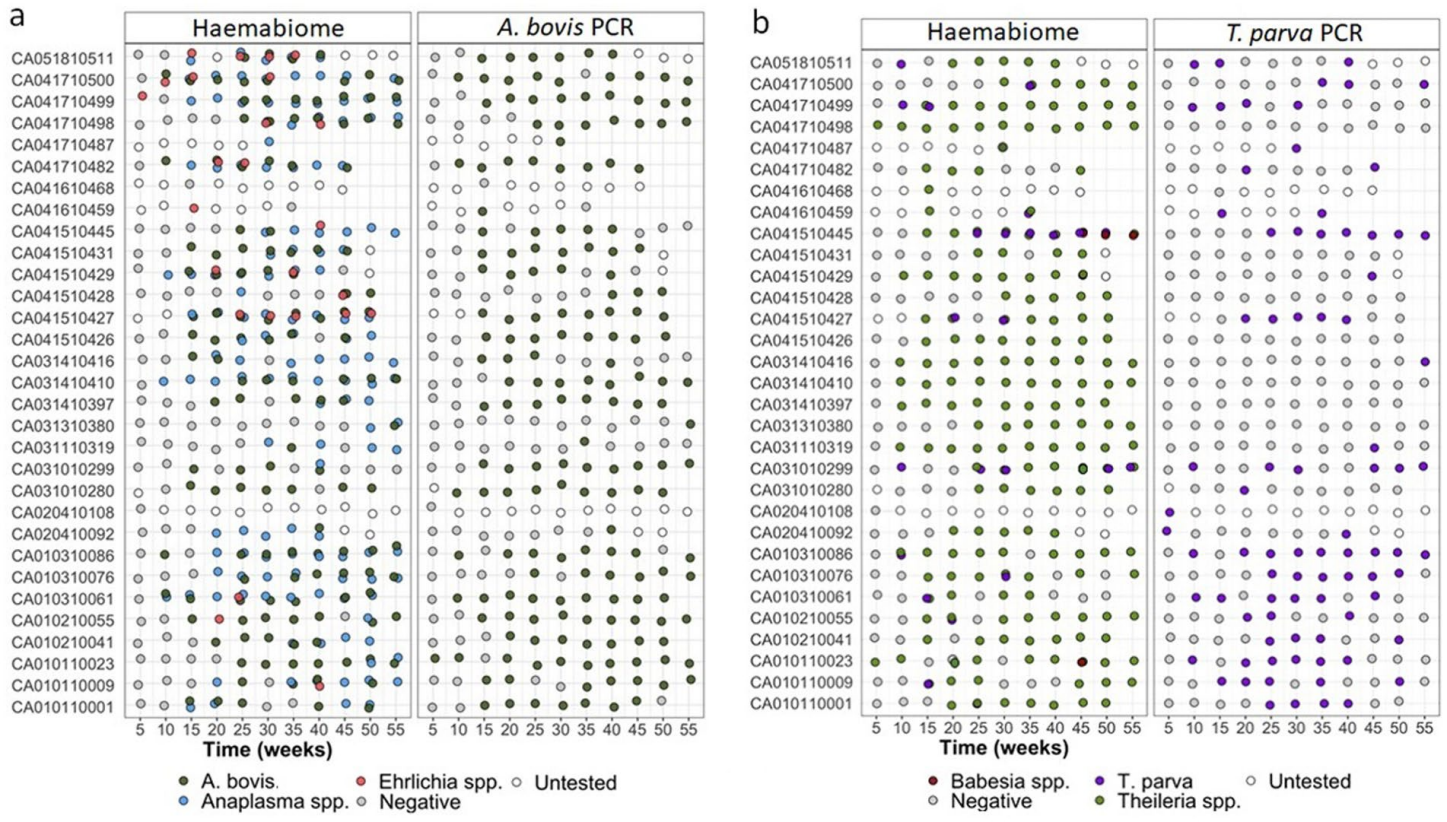
**(A)** AnEh. diagnosis of IDEAL calves across the one-year study period pathogens using different diagnostic tests across all NGS runs. Light blue dots show calf was positive any *Anaplasma* spp. infection. Orange dots present any *Ehrlichia* spp. infection. Light green dots for both. *A. bovis* infection presented in dark green dots. Negative samples (grey dots) or not tested samples (white dots) at a given 5-weekly visit during the study period using three different diagnosis approaches. **(B)** ThBa diagnosis of IDEAL calves across the one-year study period pathogens using different diagnostic tests across all NGS runs. Light green dots show calf was positive any *Theileria* spp. infection. Brown dots present any *Babesia* spp. infection. Orange dots for both. *T. parva* infection presented in purple dots. Negative samples (grey dots) or not tested samples (white dots) at a given 5-weekly visit during the study period using three different diagnosis approaches.

Figure 4 shows a comparison of the Haemabiome tool with species-specific PCR per calf and at different sampling times to confirm if the same sample is positive or negative for both approaches. This figure clearly demonstrates that samples detected as positive by the gold standard method were almost all positive by Miseq (Figures 4A,B). Additionally, the results obtained from the field samples illustrate the power of the Haemabiome tool in accurately identifying the multiplicity of infections.

### 3.2 Gel electrophoresis analysis

Initially all 279 samples were assessed by gel electrophoresis for presence of visible bands after the first round of PCR. Out of these, 108 displayed the expected band on the gel for AnEh, of which 89 were also positive for on the Miseq sequencing results, while the remaining 19 samples were negative. By comparison, 100 of the 171 AnEh samples that did not exhibit the anticipated band on gel electrophoresis, were positive for AnEh in the Miseq data, despite the lack of visible band in gel electrophoresis. Out of the 279 samples tested using the ThBa PCR 156 were positive based on gel electrophoresis, with 146 of those samples confirmed positive on Miseq sequencing. However, among the 123 samples classified as negative by gel electrophoresis, 73 were identified as positive for ThBa infection using the Haemabiome tool (Supplementary Table S5).

### 3.3 Overall diversity of *Anaplasma* and *Ehrlichia* identified by the Haemabiome tool in the IDEAL cohort field samples

Among the detected *Anaplasma* species, *A. bovis* was found in 49.5% (138/279) of samples, and *A. platys* in 42.6% (119/279), making them the most abundant species in this sample set. Uncultured *Anaplasma* sp. Saso was the third most detected species (25.1%; 70/279) followed by *Candidatus Anaplasma boleense, A. phagocytophilum* (1.4%; 4/279) and Uncultured *Anaplasma* sp. and *Anaplasma* sp. clone ZJ06 (1%, 3/279). In terms of *Ehrlichia*, *E. minasensis* (or *E. canis*) was the most abundant species, detected in 6.4%, (18/279) of samples, followed by *E. ruminantium* at 2.5% (7/279). Overall, 67.7% (189/279) of samples were positive for any *Anaplasma*/*Ehrlichia* infection (Table 5).

**Table 5:** Summary of detected hemopathogen species composition in Haemabiome tool from three group of field samples.

Nbr of animals	Nbr of samples	Genus	Species	CES		WCES		Trypanosome		Total	
				Nbr of positive samples	Frequency of infection (%95 CI)	Nbr of positive samples	Frequency of infection (%95 CI)	Nbr of positive samples	Frequency of infection (%95 CI)	Nbr of positive samples	Frequency of infection (%95 CI)
31	279	AnEh	*A. bovis*	50	53.7 (43.7–63.5)	37	39.7 (30.4–50)	51	54.8 (44.7–64.5)	138	49.5 (43.6–55)
			*Candidatus* Anaplasma boleense	4	4.3 (1.6–10.5)	0	0	0	0	4	1.4 (0.5–3.6)
			Uncultured *Anaplasma* sp. Saso	18	19.3 (12.6–28.5)	21	22.5 (15.3–32)	31	33.3 (24.6–43.4)	70	25.1 (20–30.5)
			Uncultured *Anaplasma* sp.	0	0	3	3.2 (1.1–9)	0	0	3	1 (0.3–3.1)
			*A. platys*	44	47.3 (37.4–57.3)	27	29 (20.8–38.9)	48	51.6 (41.6–61.5)	119	42.6 (36.8–48.7)
			*A. phagocytophilum*	0	0	0	0	4	4.3 (1.7–10.5)	4	1.4 (0.5–3.6)
			*E. minasensis*/*E. canis*	2	2.1 (0.6–7.5)	2	2.1 (0.6–7.5)	14	15 (9.1–23.6)	18	6.4 (4.1–9.9)
			*E. ruminatum*	1	1 (0.1–5.8)	0	0	6	6.4 (3–13.4)	7	2.5 (1.2–5.1)
			*All Anaplasma*/*Ehrlichia* sp.	66	**70.9 (61–79.2)**	55	**59.1 (48.9–68.6)**	68	**73.1 (63.3–81)**	189	67.7 (62–72.9)
		ThBa	*T. mutans*	63	67.7 (57.7–76.4)	63	67.7 (57.7–76.4)	64	68.8 (58.8–77.3)	190	68.1 (62.4–73)
			*Theileria* sp. strain MSD	45	48.4 (38.5–58.4)	45	48.4 (38.5–58.4)	52	57 (46.8–66.6)	142	50.9 (45–56.7)
			*T. parva*	6	6.4 (3–13.4)	3	3.2 (1.1–9)	14	15 (9.2–23.7)	23	8.2 (5.5–12.1)
			*T. taurotragi*	12	12.9 (7.5–21.2)	2	2.1 (0.6–7.5)	6	6.4 (3–13.4)	20	7.2 (4.7–10.8)
			*T. velifera*	34	36.5 (27.5–46.7)	28	30.1 (21.7–40)	37	39.7 (30.4–49.9)	99	35.5 (30–41.3)
			*B. bigemina*	0	0	0	0	3	3.2 (1.1–9)	3	1.1 (0.3–3.1)
			*B. bovis*	1	1 (0.1–5.8)	0	0	0	0	1	0.3 (0.6–2)
			*All Theileria*/*Babesia* sp.	75	66.6 (56.5–75.9)	74	79.5 (70.2–86.5)	70	75.3 (65.6–82.9)	219	78.5 (73.3–83)

Proportion of samples positive (±95% CI) observed when targeting the 16S/18S rRNA region. CES: experienced clinical episodes but survived. WCES: without any clinical episodes but survived. Trypanosome: animals previously diagnosed to be infected with trypanosomes.

**Figure 5:**
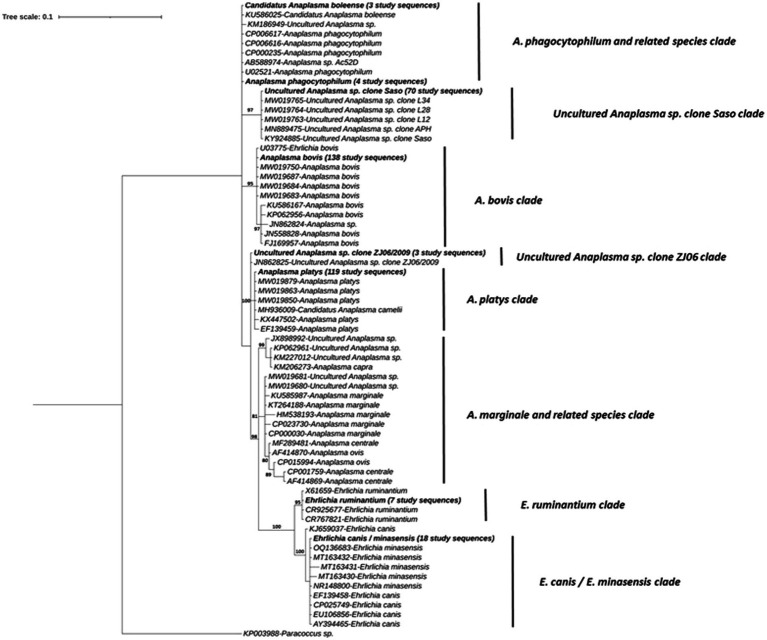
Phylogenetic tree of *Anaplasma* and *Ehrlichia* spp. determined in the subset of 31 IDEAL animals. Relationships were inferred from phylogenetic analysis of sequence data for a ~132-bp region of the 16S rRNA gene by Bayesian inference. Sequences obtained in this study are highlighted in bold with the number of samples with a specific pathogen sequence identified, indicated in brackets. Numbers at the nodes indicate % bootstrap support and the scale bar represents 0.1 substitutions per site.

The sequences from the 138 Miseq positive samples within the *Anaplasma* genus resolved into 7 distinct clades (Figure 5). The first *Anaplasma* clade consisted of all *A. phagocytophilum*, *A. phagocytophilum* related and *Candidatus Anaplasma boleense* sequences. This included three sequences from the present study that shared 100% similarity with a previously published *Candidatus Anaplasma boleense* isolate (GenBank accession, KU586025) and four sequences with previously published *A. phagocytophilum* sequences (i.e., GenBank accession, CP006617, U02521).

The second clade is represented by 70 sequences obtained from samples that share 100% nucleotide identity with *Uncultured Anaplasma* sp. *Saso* strain (GenBank accession, KY924885), including sequences previously isolated from Kenya by Okal et al. (62). The third clade comprised a number of *A. bovis* isolates with 138 (i.e., all those positive on the AnEh PCR) sequences from this study, with 100% identity to a published *A. bovis* from Okal et al. (62) in Kenya (GenBank accession, U03775). A small clade with three identical study sequences were also 100% identical to a sequence of *Uncultured Anaplasma* sp. clone ZJ06 strain (GenBank accession, JN862825) isolated from a cow in China.

A fifth clade consisted of 119 study sequences, which had previously been confirmed as *A. platys* or *A. platys-like* sequences. This group included isolates from the study conducted by Okal et al. (62) (GenBank accession, MW019850 and MW019879), as well as a *Candidatus Anaplasma camelii* sequence (GenBank accession, MH936009) derived from a Kenyan camel. Obtained sequences shared 100% nucleotide identity and 99.4% identity with other *A. platys* sequences (GenBank accession, KX447502 and EF139459), respectively.

A sixth clade was composed of *Anaplasma* species that were not detected in our field samples, including *A. marginale*, *A. centrale*, *A. ovis* and other *uncultured Anaplasma species*. A seventh clade comprised previously published *E. ruminantium* sequences, along with 7 sequences from present study. These sequences grouped into the clade with 100% sequence similarity with the counterparts in GenBank (CR767821, CR925677, X61659). Finally, the last clade encompassed the *E. canis/E. minasensis* group, which included 18 study sequences. These sequences demonstrated 100% sequence identity with counterparts in the database from different hosts (GenBank accession, AY394465, CP025749, EF139458, and EU106856 for *E. canis* and OQ136683, MT163432 and NR148800 for *E. minasensis*), and >99% of identity with the other published sequences (Figure 5).

### 3.4 Overall diversity of *Theileria* and *Babesia* identified by the Haemabiome tool in the IDEAL cohort field samples

*T. mutans* was the most abundant species and detected in 68.1% (190/279) of samples, followed by *Theileria* sp. *strain MSD* in 50.9% (142/279) of samples. *T. velifera* was the third most detected species at 35.5% (99/279). Finally, *T. parva* and *T. taurotragi* were detected in 8.2% (23/279) and 7.2% (20/279) of samples, respectively. In contrast to the high detection rate of *Theileria* species, *B. bigemina* was found in only 1.1% (3/279) and *B. bovis* in only 0.3% (1/279) of samples. Overall, 78.5% (219/279) of the samples were identified as hosting one or more *Theileria* of *Babesia* species using the Miseq sequencing approach (Table 5).

**Figure 6:**
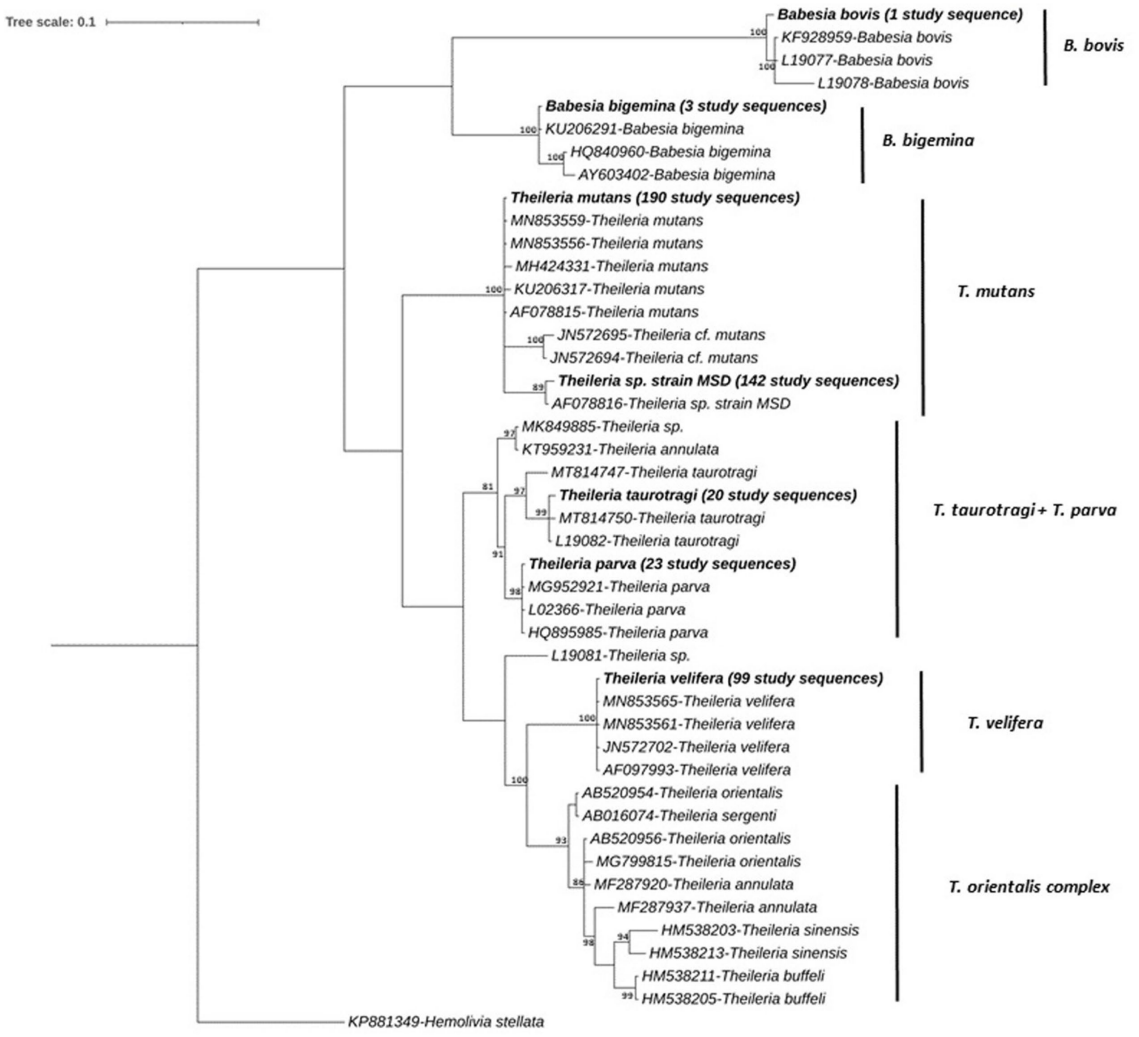
Phylogenetic relationship among consensus sequences of *Theileria* and *Babesia.* spp. determined in IDEAL subset animals. Relationships were inferred from phylogenetic analysis of sequence data for a ~405-bp region of the 18S rRNA gene by Bayesian inference. Sequences obtained in this study are highlighted in bold with the number of samples with specific pathogen sequence identified indicated in brackets. Numbers at the nodes indicate % bootstrap support and the scale bar represents 0.1 substitutions per site.

The *Theileria* and *Babesia* sequences could be grouped into five clades, and the phylogenetic tree is shown in Figure 6. The first clade contained only an unique sequence from this study, detected in a single sample, which clustered with a *B. bovis* published sequence (GenBank accession, L19077 and KF928959) with >99.1% nucleotide identity. The second clade included one sequence identified in three samples this study, which had 100% nucleotide similarity with a *B. bigemina* sequence isolated from zebu cattle in Uganda (GenBank accession, KU206291) as reported by Byaruhanga et al. (63).

A third clade encompassed one sequence which was detected in 190 study samples, which grouped together with 100% nucleotide identity with previously published *T. mutans* sequences (GenBank accession, MN853559 and MN853556); >99.7% identity was observed with other published sequences, and another sequence which was detected in 142 study samples grouped with *Theileria* sp. *strain MSD* (99.7% identity, GenBank accession, AF078816). A fourth clade contained three distinct sequences, detected in 20 study samples. The first sequence was detected in four samples and showed 99.69% nucleotide identity with published *T. taurotragi* sequences (GenBank accessions MN744953 and L19082). The second sequence was detected in 14 samples and showed 100 and 99.73% nucleotide identity with published *T. taurotragi* sequences (GenBank accessions MN744953 and L19082, respectively). The third sequence was found in two samples and showed 99.38 and 99.07% nucleotide identity with published *T. taurotragi* sequences (GenBank accessions L19082 and MT814750, respectively).

Furthermore, a *T. parva* sequence was detected in 23 study samples, sharing 99.7% nucleotide identity with the published sequences (GenBank accession, MG952921 and HQ895985). The final clade contained a *T. velifera* sequence, detected in 99 study samples, forming a distinct group with 100% identity with counterparts in the database (Genbank accession, AF097993, JN572702, MN853561 and MN853565). *Theileria* species such as *T. annulata*, *T. buffeli, T. orientalis and T. sinensis* presented in a separate group, although no sequences from this study aligned with these species (Figure 6).

## 4 Discussion

In the IDEAL project, Bronsvoort et al. (8) quantified for the first time in an animal population the high diversity of pathogens that a population may have to deal with over time (the first year of life), and the levels of co-infections with key pathogens such as *A. marginale*, *E. ruminantium* and *T. parva*. They also highlighted the need to develop new systems-based approaches to study pathogens in their natural settings, in order to understand the impacts of co-infections on clinical outcomes and to develop new evidence-based interventions that are relevant. The preliminary presentation of the pathogens has hitherto been simply summed across all visits to estimate the proportion of calves with each pathogen (or pathogen/test combination). However, this ignores the dynamics of the order of exposure.

The current reanalysis of a subset of these samples with the novel Haemabiome tool has demonstrated that the diversity of co-infections at any point can be resolved sufficiently to allow more detailed co-infection studies, which require a high-throughput analysis of the pathogen dynamics and distribution. The Haemabiome tool appears to have very high specificity but moderate sensitivity compared to the two nested PCRs used as the gold standard in this dataset. However, this is to be expected given the very high levels of specificity and sensitivity in the nested PCRs used. The nested PCRs use 60 cycles, whereas only 35 cycles were used to generate the sequencing library. Additionally, it has been suggested by previous studies that NGS approaches may not be ideal for absolute quantification. The more limited sensitivity of NGS datasets can be attributed to potential factors such as mixed infections, PCR competition, and PCR suppression caused by the presence of more abundant templates (32). There is an obvious trade-off in terms of reduced sensitivity with the high throughput nature and ability of the Haemabiome tool to detect the full range of pathogen diversity in the target genera. For example, animals that are carriers for *T. parva* (a relatively common state of infection, particularly in adult animals) have low levels of parasitaemia, which might fall under the detection level of the Haemobiome tool.

The diversity of tick-borne pathogen communities detected using the Haemabiome tool is well supported by previous studies conducted in western Kenya on cattle from various genus specific PCR (57, 62, 64). For *Anaplasma*, *A. bovis* and *A. platys* were the most abundant species in both our and the previous studies. However, in our study we did not find any *A. marginale* infections, while the studies noted above detected *A. marginale* infection, albeit at low prevalence: only 0.6% in Okal et al. (62) and 4.9% in Chiuya et al. (57), respectively whereas Peter et al. (64) detected 31% of *A. marginale* infections. Okal et al. (62) did not find any *Ehrlichia* spp. and Chiuya et al. (57) only identified *E. minasensis*. *Ehrlichia* sequences amplified from samples in our study cluster phylogenetically between *E. canis* and *E. minasensis* reference sequences. As *E. minasensis* is a tick-borne pathogen affecting cattle, cervids, and dogs, and is closely related to the monocytotropic pathogen *E. canis* (65), without further investigation it is only possible to classify samples in our study as *E. canis*/*E. minasensis*, highlighting the need for a more accurately curated and appropriately diverse reference sequence database in this approach. Further studies are therefore needed to better understand the epidemiology and dynamics of *Ehrlichia* transmission in livestock in western Kenya. We detected most *Theileria* species that were previously identified in western Kenya, and *T. mutans* and *T. velifera* being the most abundant *Theileria* species is consistent with previous studies in this region (62, 63, 66). Additionally, we confirmed that the important cattle pathogen *T. parva* is circulating in this area. We did not find substantial levels of *Babesia* spp. infection, but overall, our findings are consistent with previous studies confirming theileriosis is the major circulating infectious piroplasm disease of cattle in western Kenya (57, 62, 66).

Bronsvoort et al. (8) highlighted that livestock disease and vector control are indispensable for increasing livestock productivity and preventing losses due to diseases, including disease-related morbidity, mortality, and loss of markets for livestock products in Africa. The lack of disease control measures has implications for the effectiveness of strategies aimed at rapidly improving livelihoods dependent on livestock. One route to improving disease control is to increase the accuracy of identification of infected animals, in order to apply targeted treatment. However, this approach is economically viable only if a single assay can detect as many pathogens as possible. Conducting multiple single-pathogen tests is not economically or logistically feasible, particularly in settings where co-infections are the norm. Current diagnostic markers are species or genus specific and difficult (if not impossible) to scale up in a high-throughput manner.

In this study, we have designed a deep amplicon sequencing tool that in principle will enable detection to investigate the species composition of tick-borne pathogens present in cattle. The Haemabiome tool has the power to provide more accurate and reliable quantification of the pathogen community in terms of species diversity, but can also detect previously unanticipated (or untargeted) pathogen species that may play a role in animal disease.

Overall, we describe the development of a high-throughput amplicon sequencing approach targeting tick-borne pathogen genuses of high relevance to cattle health (*Anaplasma*, *Ehrlichia*, *Theileria* and *Babesia* species). The tool permitted us to identify the full diversity of tick-borne pathogens circulating, and its reliability and utility was demonstrated on field samples for which data for particular pathogens was already available. The Haemabiome tool will allow us to explore a range of epidemiological questions in the future, through the generation of large-scale data on the diversity of tick-borne pathogens at a population level. Improved understanding of the diversity of pathogen infections encountered, and the potential impact on disease outcome or severity of interactions between such pathogens, will enable a more efficient combating of the multiple infectious disease threats in the African smallholder farm context, with vector-borne diseases amongst the most impactful.

## Data Availability

The datasets presented in this study can be found in online repositories. The names of the repository/repositories and accession number(s) can be found at: https://www.ebi.ac.uk/ena, PRJEB79313.

## Footnotes

^1^ https://ngphylogeny.fr
