# Supplementary material for: Development of a novel Haemabiome tool for the high-throughput analysis of haemopathogen species co-infections in African livestock
Source: Front Vet Sci. 2024 Dec 20;11:1491828. doi: 10.3389/fvets.2024.1491828 (PMC11695320; doi:10.3389/fvets.2024.1491828)
Supplement: Supplementary file 1 [file Table_1.DOCX]

**Development of a novel haemabiome tool for the high-throughput analysis of haemopathogen species co-infections in African livestock**

**Supplementary information:**

**Yalcindag et al.**

**Supplementary Table S1:** Characteristics of the calves blood samples used and sequenced in this study with, individual Calf ID, collection dates and visit ID, animal life status, clinical episode status, sample ID and animal categories. NA: Not applicable. CES: experienced clinical episodes but survived. WCES: without any clinical episodes but survived. TRYPS: animals previously diagnosed to be infected with trypanosomes. * 8 samples were repeatedly sequenced into 2 different runs.

|  |  |  |  |  |  |  |
| --- | --- | --- | --- | --- | --- | --- |
| **CalfID** | **VisitID** | **VisitDate** | **Dead / Alive** | **Clinical Episode survived** | **SampleID** | **Animal categories** |
| CA010110001 | VRC010001 | 10/10/2007 | Alive | Yes | RED000008 | CES |
|  | VRC060001 | 14/11/2007 | Alive | Yes | RED000068 | CES |
|  | VRC110001 | 19/12/2007 | Alive | Yes | RED000133 | CES |
|  | VRC160001 | 23/01/2008 | Alive | Yes | RED000192 | CES |
|  | VRC260001 | 02/04/2008 | Alive | Yes | RED000254 | CES |
|  | VRC310001 | 07/05/2008 | Alive | Yes | RED000556 | CES |
|  | VRC360001 | 11/06/2008 | Alive | Yes | RED000672 | CES |
|  | VRC410001 | 16/07/2008 | Alive | Yes | RED000930 | CES |
|  | VRC460001 | 20/08/2008 | Alive | Yes | RED001336 | CES |
|  | VRC510001 | 24/09/2008 | Alive | Yes | RED001664* | CES |
| CA010110009 | VRC010009 | 03/04/2008 | Alive | Yes | RED000444 | CES |
|  | VRC060009 | 07/05/2008 | Alive | Yes | RED000562 | CES |
|  | VRC110009 | 11/06/2008 | Alive | Yes | RED000678 | CES |
|  | VRC160009 | 16/07/2008 | Alive | Yes | RED000938 | CES |
|  | VRC210009 | 20/08/2008 | Alive | Yes | RED001343 | CES |
|  | VRC260009 | 24/09/2008 | Alive | Yes | RED001748 | CES |
|  | VRC310009 | 29/10/2008 | Alive | Yes | RED001882 | CES |
|  | VRC360009 | 03/12/2008 | Alive | Yes | RED002315 | CES |
|  | VRC410009 | 07/01/2009 | Alive | Yes | RED002488* | CES |
|  | VRC460009 | 11/02/2009 | Alive | Yes | RED002795* | CES |
|  | VRC510009 | 18/03/2009 | Alive | Yes | RED003204* | CES |
| CA010110023 | VRC010023 | 22/04/2009 | Alive | Yes | RED003327 | CES |
|  | VRC060023 | 27/05/2009 | Alive | Yes | RED003700 | CES |
|  | VRC110023 | 01/07/2009 | Alive | Yes | RED004130 | CES |
|  | VRC160023 | 05/08/2009 | Alive | Yes | RED004648 | CES |
|  | VRC210023 | 09/09/2009 | Alive | Yes | RED005001 | CES |
|  | VRC260023 | 14/10/2009 | Alive | Yes | RED005240 | CES |
|  | VRC310023 | 18/11/2009 | Alive | Yes | RED005530 | CES |
|  | VRC360023 | 20/12/2009 | Alive | Yes | RED005747 | CES |
|  | VRC410023 | 27/01/2010 | Alive | Yes | RED005891 | CES |
|  | VRC460023 | 03/03/2010 | Alive | Yes | RED006108 | CES |
|  | VRC510023 | 07/04/2010 | Alive | Yes | RED006285 | CES |
| CA010210041 | VRC010041 | 03/07/2008 | Alive | Yes | RED000884 | CES |
|  | VRC060041 | 06/08/2008 | Alive | Yes | RED000996 | CES |
|  | VRC110041 | 10/09/2008 | Alive | Yes | RED001494 | CES |
|  | VRC160041 | 15/10/2008 | Alive | Yes | RED001925 | CES |
|  | VRC210041 | 19/11/2008 | Alive | Yes | RED002161 | CES |
|  | VRC310041 | 28/01/2009 | Alive | Yes | RED002662 | CES |
|  | VRC360041 | 04/03/2009 | Alive | Yes | RED003158 | CES |
|  | VRC410041 | 08/04/2009 | Alive | Yes | RED003291 | CES |
|  | VRC460041 | 13/05/2009 | Alive | Yes | RED003723 | CES |
|  | VRC510041 | 17/06/2009 | Alive | Yes | RED004055 | CES |
| CA010210055 | VRC010055 | 23/07/2009 | Alive | Yes | RED004406 | CES |
|  | VRC060055 | 26/08/2009 | Alive | Yes | RED004840 | CES |
|  | VRC110055 | 30/09/2009 | Alive | Yes | RED005158 | CES |
|  | VRC160055 | 04/11/2009 | Alive | Yes | RED005401 | CES |
|  | VRC210055 | 09/12/2009 | Alive | Yes | RED005629 | CES |
|  | VRC260055 | 13/01/2010 | Alive | Yes | RED005845 | CES |
|  | VRC310055 | 17/02/2010 | Alive | Yes | RED006066 | CES |
|  | VRC360055 | 24/03/2010 | Alive | Yes | RED006211 | CES |
|  | VRC410055 | 28/04/2010 | Alive | Yes | RED006481 | CES |
|  | VRC460055 | 02/06/2010 | Alive | Yes | RED006352 | CES |
|  | VRC510055 | 07/07/2010 | Alive | Yes | RED006665 | CES |
| CA010310061 | VRC010061 | 22/10/2007 | Alive | NA | RED000019 | CES |
|  | VRC060061 | 28/11/2007 | Alive | NA | RED000108 | CES |
|  | VRC110061 | 09/01/2008 | Alive | NA | RED000159 | CES |
|  | VRC210061 | 19/03/2008 | Alive | NA | RED000219 | CES |
|  | VRC260061 | 16/04/2008 | Alive | NA | RED000292 | CES |
|  | VRC310061 | 21/05/2008 | Alive | NA | RED000742 | CES |
|  | VRC360061 | 25/06/2008 | Alive | NA | RED000775 | CES |
|  | VRC410061 | 30/07/2008 | Alive | NA | RED000976 | CES |
|  | VRC460061 | 03/09/2008 | Alive | NA | RED001388 | CES |
|  | VRC510061 | 11/10/2008 | Alive | NA | RED001811 | CES |
| CA010310076 | VRC010076 | 13/01/2009 | Alive | NA | RED002529 | CES |
|  | VRC060076 | 25/02/2009 | Alive | NA | RED002859 | CES |
|  | VRC110076 | 01/04/2009 | Alive | NA | RED003077 | CES |
|  | VRC160076 | 06/05/2009 | Alive | NA | RED003592 | CES |
|  | VRC210076 | 10/06/2009 | Alive | NA | RED004026 | CES |
|  | VRC260076 | 15/07/2009 | Alive | NA | RED004200 | CES |
|  | VRC310076 | 19/08/2009 | Alive | NA | RED004697 | CES |
|  | VRC360076 | 23/09/2009 | Alive | NA | RED005128 | CES |
|  | VRC410076 | 28/10/2009 | Alive | NA | RED005366 | CES |
|  | VRC460076 | 02/12/2009 | Alive | NA | RED005600 | CES |
|  | VRC510076 | 06/01/2010 | Alive | NA | RED005770 | CES |
| CA010310086 | VRC010086 | 14/09/2009 | Alive | NA | RED004911 | CES |
|  | VRC060086 | 28/10/2009 | Alive | NA | RED005371 | CES |
|  | VRC110086 | 02/12/2009 | Alive | NA | RED005604 | CES |
|  | VRC160086 | 06/01/2010 | Alive | NA | RED005821 | CES |
|  | VRC210086 | 10/02/2010 | Alive | NA | RED005942 | CES |
|  | VRC260086 | 17/03/2010 | Alive | NA | RED006251 | CES |
|  | VRC310086 | 21/04/2010 | Alive | NA | RED006447 | CES |
|  | VRC360086 | 26/05/2010 | Alive | NA | RED006339 | CES |
|  | VRC410086 | 30/06/2010 | Alive | NA | RED006639 | CES |
|  | VRC460086 | 02/08/2010 | Alive | NA | RED006703 | CES |
|  | VRC510086 | 08/09/2010 | Alive | NA | RED006710 | CES |
| CA020410092 | VRC010092 | 17/10/2007 | Alive | NA | RED000014 | CES |
|  | VRC060092 | 21/11/2007 | Alive | NA | RED000101 | CES |
|  | VRC110092 | 22/12/2007 | Alive | NA | RED000142 | CES |
|  | VRC210092 | 21/03/2008 | Alive | NA | RED000231 | CES |
|  | VRC260092 | 09/04/2008 | Alive | NA | RED000276 | CES |
|  | VRC310092 | 14/05/2008 | Alive | NA | RED000580 | CES |
|  | VRC360092 | 18/06/2008 | Alive | NA | RED000746 | CES |
|  | VRC410092 | 23/07/2008 | Alive | NA | RED000954 | CES |
| CA031010280 | VRC060280 | 03/07/2008 | Alive | No | RED000892 | WCES |
|  | VRC110280 | 07/08/2008 | Alive | No | RED000999 | WCES |
|  | VRC160280 | 11/09/2008 | Alive | No | RED001619 | WCES |
|  | VRC210280 | 16/10/2008 | Alive | No | RED001927* | WCES |
|  | VRC260280 | 20/11/2008 | Alive | No | RED002166 | WCES |
|  | VRC360280 | 29/01/2009 | Alive | No | RED002755 | WCES |
|  | VRC410280 | 05/03/2009 | Alive | No | RED003167 | WCES |
|  | VRC460280 | 09/04/2009 | Alive | No | RED003299 | WCES |
|  | VRC510280 | 14/05/2009 | Alive | No | RED003726 | WCES |
| CA031010299 | VRC010299 | 23/09/2009 | Alive | No | RED004942 | WCES |
|  | VRC060299 | 05/11/2009 | Alive | No | RED004977 | WCES |
|  | VRC110299 | 10/12/2009 | Alive | No | RED005714 | WCES |
|  | VRC160299 | 14/01/2010 | Alive | No | RED005847 | WCES |
|  | VRC210299 | 18/02/2010 | Alive | No | RED005996 | WCES |
|  | VRC260299 | 25/03/2010 | Alive | No | RED006323 | WCES |
|  | VRC310299 | 29/04/2010 | Alive | No | RED006485 | WCES |
|  | VRC360299 | 03/06/2010 | Alive | No | RED006355 | WCES |
|  | VRC410299 | 08/07/2010 | Alive | No | RED006668 | WCES |
|  | VRC460299 | 12/08/2010 | Alive | No | RED006854 | WCES |
|  | VRC510299 | 16/09/2010 | Alive | No | RED006899 | WCES |
| CA031110319 | VRC010319 | 23/03/2009 | Alive | No | RED002978 | WCES |
|  | VRC060319 | 16/04/2009 | Alive | No | RED003523 | WCES |
|  | VRC110319 | 21/05/2009 | Alive | No | RED003752 | WCES |
|  | VRC160319 | 25/06/2009 | Alive | No | RED004087 | WCES |
|  | VRC210319 | 30/07/2009 | Alive | No | RED004627 | WCES |
|  | VRC260319 | 03/09/2009 | Alive | No | RED004871* | WCES |
|  | VRC310319 | 08/10/2009 | Alive | No | RED005197 | WCES |
|  | VRC360319 | 12/11/2009 | Alive | No | RED005445* | WCES |
|  | VRC410319 | 17/12/2009 | Alive | No | RED005667* | WCES |
|  | VRC460319 | 21/01/2010 | Alive | No | RED004265 | WCES |
|  | VRC510319 | 25/02/2010 | Alive | No | RED006096 | WCES |
| CA031310380 | VRC010380 | 11/02/2009 | Alive | No | RED002588 | WCES |
|  | VRC060380 | 17/03/2009 | Alive | No | RED003023 | WCES |
|  | VRC110380 | 21/04/2009 | Alive | No | RED003454 | WCES |
|  | VRC160380 | 26/05/2009 | Alive | No | RED003693 | WCES |
|  | VRC210380 | 30/06/2009 | Alive | No | RED004124 | WCES |
|  | VRC260380 | 04/08/2009 | Alive | No | RED004642 | WCES |
|  | VRC310380 | 08/09/2009 | Alive | No | RED004796 | WCES |
|  | VRC360380 | 13/10/2009 | Alive | No | RED005233 | WCES |
|  | VRC410380 | 17/11/2009 | Alive | No | RED005525 | WCES |
|  | VRC460380 | 19/12/2009 | Alive | No | RED005742 | WCES |
|  | VRC510380 | 26/01/2010 | Alive | No | RED004281 | WCES |
| CA031410397 | VRC010397 | 02/06/2008 | Alive | No | RED000818 | WCES |
|  | VRC060397 | 01/07/2008 | Alive | No | RED000793 | WCES |
|  | VRC110397 | 05/08/2008 | Alive | No | RED001185 | WCES |
|  | VRC160397 | 09/09/2008 | Alive | No | RED001491 | WCES |
|  | VRC210397 | 14/10/2008 | Alive | No | RED001825 | WCES |
|  | VRC260397 | 18/11/2008 | Alive | No | RED002245 | WCES |
|  | VRC360397 | 27/01/2009 | Alive | No | RED002744 | WCES |
|  | VRC410397 | 03/03/2009 | Alive | No | RED003152 | WCES |
|  | VRC460397 | 07/04/2009 | Alive | No | RED003281 | WCES |
|  | VRC510397 | 12/05/2009 | Alive | No | RED003714 | WCES |
| CA031410410 | VRC010410 | 07/04/2009 | Alive | No | RED002992 | WCES |
|  | VRC060410 | 12/05/2009 | Alive | No | RED003636 | WCES |
|  | VRC110410 | 16/06/2009 | Alive | No | RED003969 | WCES |
|  | VRC160410 | 21/07/2009 | Alive | No | RED004507 | WCES |
|  | VRC210410 | 25/08/2009 | Alive | No | RED004745 | WCES |
|  | VRC260410 | 29/09/2009 | Alive | No | RED005070 | WCES |
|  | VRC310410 | 03/11/2009 | Alive | No | RED004975 | WCES |
|  | VRC360410 | 08/12/2009 | Alive | No | RED005707 | WCES |
|  | VRC410410 | 12/01/2010 | Alive | No | RED005791 | WCES |
|  | VRC460410 | 16/02/2010 | Alive | No | RED005960 | WCES |
|  | VRC510410 | 23/03/2010 | Alive | No | RED006259 | WCES |
| CA031410416 | VRC010416 | 24/09/2009 | Alive | No | RED004944 | WCES |
|  | VRC060416 | 03/11/2009 | Alive | No | RED004971 | WCES |
|  | VRC110416 | 08/12/2009 | Alive | No | RED005705 | WCES |
|  | VRC160416 | 12/01/2010 | Alive | No | RED005794 | WCES |
|  | VRC210416 | 16/02/2010 | Alive | No | RED005964 | WCES |
|  | VRC260416 | 23/03/2010 | Alive | No | RED006319 | WCES |
|  | VRC310416 | 27/04/2010 | Alive | No | RED006473 | WCES |
|  | VRC360416 | 01/06/2010 | Alive | No | RED006360 | WCES |
|  | VRC410416 | 06/07/2010 | Alive | No | RED006663 | WCES |
|  | VRC460416 | 10/08/2010 | Alive | No | RED006850 | WCES |
|  | VRC510416 | 14/09/2010 | Alive | No | RED006719 | WCES |
| CA041510426 | VRC010426 | 28/04/2008 | Alive | No | RED000488 | WCES |
|  | VRC060426 | 02/06/2008 | Alive | No | RED000638 | WCES |
|  | VRC110426 | 07/07/2008 | Alive | No | RED000901 | WCES |
|  | VRC160426 | 11/08/2008 | Alive | No | RED001304 | WCES |
|  | VRC210426 | 15/09/2008 | Alive | No | RED001625 | WCES |
|  | VRC260426 | 20/10/2008 | Alive | No | RED001941 | WCES |
|  | VRC310426 | 24/11/2008 | Alive | No | RED002172 | WCES |
|  | VRC410426 | 02/02/2009 | Alive | No | RED002760 | WCES |
|  | VRC460426 | 09/03/2009 | Alive | No | RED003169 | WCES |
|  | VRC510426 | 13/04/2009 | Alive | No | RED003502 | WCES |
| CA041510427 | VRC110427 | 07/07/2008 | Alive | NA | RED000904 | TRYPS |
|  | VRC160427 | 11/08/2008 | Alive | NA | RED001401 | TRYPS |
|  | VRC210427 | 15/09/2008 | Alive | NA | RED001628 | TRYPS |
|  | VRC260427 | 20/10/2008 | Alive | NA | RED001842 | TRYPS |
|  | VRC310427 | 24/11/2008 | Alive | NA | RED002267 | TRYPS |
|  | VRC410427 | 02/02/2009 | Alive | NA | RED002671 | TRYPS |
|  | VRC460427 | 09/03/2009 | Alive | NA | RED002897 | TRYPS |
|  | VRC510427 | 13/04/2009 | Alive | NA | RED003503 | TRYPS |
| CA041510428 | VRC010428 | 02/06/2008 | Alive | NA | RED000816 | TRYPS |
|  | VRC060428 | 07/07/2008 | Alive | NA | RED001117 | TRYPS |
|  | VRC110428 | 11/08/2008 | Alive | NA | RED001402 | TRYPS |
|  | VRC160428 | 15/09/2008 | Alive | NA | RED001707 | TRYPS |
|  | VRC210428 | 20/10/2008 | Alive | NA | RED001938 | TRYPS |
|  | VRC260428 | 24/11/2008 | Alive | NA | RED002265 | TRYPS |
|  | VRC360428 | 02/02/2009 | Alive | NA | RED002674 | TRYPS |
|  | VRC410428 | 09/03/2009 | Alive | NA | RED002899 | TRYPS |
|  | VRC460428 | 13/04/2009 | Alive | NA | RED003426 | TRYPS |
|  | VRC510428 | 18/05/2009 | Alive | NA | RED003729 | TRYPS |
| CA041510429 | VRC010429 | 09/07/2008 | Alive | No | RED001202 | WCES |
|  | VRC060429 | 11/08/2008 | Alive | No | RED001404 | WCES |
|  | VRC110429 | 15/09/2008 | Alive | No | RED001711 | WCES |
|  | VRC160429 | 20/10/2008 | Alive | No | RED001845 | WCES |
|  | VRC210429 | 24/11/2008 | Alive | No | RED002270 | WCES |
|  | VRC310429 | 02/02/2009 | Alive | No | RED002676 | WCES |
|  | VRC360429 | 09/03/2009 | Alive | No | RED002896 | WCES |
|  | VRC410429 | 13/04/2009 | Alive | No | RED003310 | WCES |
|  | VRC460429 | 18/05/2009 | Alive | No | RED003658 | WCES |
| CA041510431 | VRC010431 | 26/08/2008 | Alive | NA | RED001292 | TRYPS |
|  | VRC060431 | 15/09/2008 | Alive | NA | RED001709 | TRYPS |
| 34ee | VRC110431 | 20/10/2008 | Alive | NA | RED001936 | TRYPS |
|  | VRC160431 | 24/11/2008 | Alive | NA | RED002266 | TRYPS |
|  | VRC260431 | 02/02/2009 | Alive | NA | RED002673 | TRYPS |
|  | VRC310431 | 09/03/2009 | Alive | NA | RED002898 | TRYPS |
|  | VRC360431 | 13/04/2009 | Alive | NA | RED003425 | TRYPS |
|  | VRC410431 | 18/05/2009 | Alive | NA | RED003661 | TRYPS |
|  | VRC460431 | 22/06/2009 | Alive | NA | RED003996 | TRYPS |
| CA041510445 | VRC010445 | 01/09/2009 | Alive | NA | RED004477 | TRYPS |
|  | VRC060445 | 05/10/2009 | Alive | NA | RED005204 | TRYPS |
|  | VRC110445 | 09/11/2009 | Alive | NA | RED005422 | TRYPS |
|  | VRC160445 | 14/12/2009 | Alive | NA | RED005646 | TRYPS |
|  | VRC210445 | 18/01/2010 | Alive | NA | RED005854 | TRYPS |
|  | VRC260445 | 22/02/2010 | Alive | NA | RED005977 | TRYPS |
|  | VRC310445 | 29/03/2010 | Alive | NA | RED006293 | TRYPS |
|  | VRC360445 | 03/05/2010 | Alive | NA | RED006508 | TRYPS |
|  | VRC410445 | 07/06/2010 | Alive | NA | RED006372 | TRYPS |
|  | VRC460445 | 12/07/2010 | Alive | NA | RED006650 | TRYPS |
|  | VRC510445 | 16/08/2010 | Alive | NA | RED006860 | TRYPS |
| CA041610459 | VCC010459 | 25/09/2008 | Alive | NA | RED001671 | TRYPS |
|  | VCC020459 | 23/12/2008 | Alive | NA | RED002469 | TRYPS |
| CA041710482 | VRC010482 | 23/10/2007 | Alive | NA | RED000024 | TRYPS |
|  | VRC060482 | 27/11/2007 | Alive | NA | RED000106 | TRYPS |
|  | VRC110482 | 08/01/2008 | Alive | NA | RED000152 | TRYPS |
|  | VRC260482 | 15/04/2008 | Alive | NA | RED000291 | TRYPS |
|  | VRC310482 | 20/05/2008 | Alive | NA | RED000602 | TRYPS |
|  | VRC360482 | 24/06/2008 | Alive | NA | RED000771 | TRYPS |
|  | VRC410482 | 29/07/2008 | Alive | NA | RED000972 | TRYPS |
|  | VRC460482 | 02/09/2008 | Alive | NA | RED001387 | TRYPS |
|  | VRC510482 | 07/10/2008 | Alive | NA | RED001809 | TRYPS |
| CA041710487 | VCC010487 | 18/11/2008 | Alive | NA | RED002155 | TRYPS |
| CA041710498 | VRC010498 | 04/02/2009 | Alive | NA | RED002572 | TRYPS |
|  | VRC060498 | 31/03/2009 | Alive | NA | RED003070 | TRYPS |
|  | VRC110498 | 05/05/2009 | Alive | NA | RED003611 | TRYPS |
|  | VRC160498 | 09/06/2009 | Alive | NA | RED003941 | TRYPS |
|  | VRC210498 | 14/07/2009 | Alive | NA | RED004197 | TRYPS |
|  | VRC260498 | 18/08/2009 | Alive | NA | RED004721 | TRYPS |
|  | VRC310498 | 22/09/2009 | Alive | NA | RED005094 | TRYPS |
|  | VRC360498 | 27/10/2009 | Alive | NA | RED005285 | TRYPS |
|  | VRC410498 | 01/12/2009 | Alive | NA | RED005570 | TRYPS |
|  | VRC460498 | 05/01/2010 | Alive | NA | RED005766 | TRYPS |
|  | VRC510498 | 09/02/2010 | Alive | NA | RED006027 | TRYPS |
| CA041710499 | VRC010499 | 18/02/2009 | Alive | NA | RED002599 | TRYPS |
|  | VRC060499 | 31/03/2009 | Alive | NA | RED003071 | TRYPS |
|  | VRC110499 | 05/05/2009 | Alive | NA | RED003588 | TRYPS |
|  | VRC160499 | 09/06/2009 | Alive | NA | RED004023 | TRYPS |
|  | VRC210499 | 14/07/2009 | Alive | NA | RED004357 | TRYPS |
|  | VRC260499 | 18/08/2009 | Alive | NA | RED004690 | TRYPS |
|  | VRC310499 | 22/09/2009 | Alive | NA | RED005123 | TRYPS |
|  | VRC360499 | 27/10/2009 | Alive | NA | RED005363 | TRYPS |
|  | VRC410499 | 01/12/2009 | Alive | NA | RED005595 | TRYPS |
|  | VRC460499 | 05/01/2010 | Alive | NA | RED005812 | TRYPS |
|  | VRC510499 | 09/02/2010 | Alive | NA | RED006028 | TRYPS |
| CA041710500 | VRC010500 | 16/03/2009 | Alive | NA | RED002961 | TRYPS |
|  | VRC060500 | 05/05/2009 | Alive | NA | RED003609 | TRYPS |
|  | VRC110500 | 09/06/2009 | Alive | NA | RED003940 | TRYPS |
|  | VRC160500 | 14/07/2009 | Alive | NA | RED004196 | TRYPS |
|  | VRC210500 | 18/08/2009 | Alive | NA | RED004720 | TRYPS |
|  | VRC260500 | 22/09/2009 | Alive | NA | RED005093 | TRYPS |
|  | VRC310500 | 27/10/2009 | Alive | NA | RED005287 | TRYPS |
|  | VRC360500 | 01/12/2009 | Alive | NA | RED005569 | TRYPS |
|  | VRC410500 | 05/01/2010 | Alive | NA | RED005767 | TRYPS |
|  | VRC460500 | 09/02/2010 | Alive | NA | RED005939 | TRYPS |
|  | VRC510500 | 16/03/2010 | Alive | NA | RED006204 | TRYPS |
| CA051810511 | VCC010511 | 21/03/2008 | Alive | NA | RED000420 | TRYPS |
|  | VRC010511 | 29/10/2007 | Alive | NA | RED000028 | TRYPS |
|  | VRC060511 | 03/12/2007 | Alive | NA | RED000112 | TRYPS |
|  | VRC110511 | 08/01/2008 | Alive | NA | RED000351 | TRYPS |
|  | VRC210511 | 17/03/2008 | Alive | NA | RED000203 | TRYPS |
|  | VRC260511 | 21/04/2008 | Alive | NA | RED000503 | TRYPS |
|  | VRC310511 | 26/05/2008 | Alive | NA | RED000612 | TRYPS |
|  | VRC360511 | 30/06/2008 | Alive | NA | RED001104 | TRYPS |
| CA041610468 | VCC010468 | 15/04/2009 | Alive | NA | RED003315 | TRYPS |
| CA020410108 | VRC010108 | 13/01/2009 | Alive | NA | RED002527 | TRYPS |
|  |  |  |  |  |  |  |

**Supplementary Table S2:** List of all sequences used in this study from Genbank for Anaplasma / Ehrlichia dataset. * Sequences used only to detect designed primers sets. ** Sequences used only for phylogenetic tree. *** Sequences used for both analysis. NA: information not available, ds: unpublished sequences that directly submitted to NCBI.

| Genbank accession number | Organism | Host | Reference |
| --- | --- | --- | --- |
| KU586167*** | *A. bovis* | *Anapheles sinensis* | (Guo *et al.* 2016) |
| KP062958* | *A. bovis* | Goat | (Ge *et al.* 2016) |
| AF318944* | *A. centrale* | NA | (Bekker *et al.* 2002) |
| MF289481*** | *A. centrale* | Cattle | (Zhou *et al.* 2018) |
| AF309867* | *A. marginale* | NA | ds |
| KU686789* | *A. marginale* | Cattle | (Byaruhanga *et al.* 2018) |
| AF414870*** | *A. ovis* | NA | (Lew *et al.* 2003) |
| AF414869** | *A. centrale* | NA |  |
| AF414877** | *A. marginale* | NA |  |
| MN266938* | *A. ovis* | Tick | (Sang *et al.* 2006) |
| U02521*** | *A. phagocytophilum* | NA | (Chen *et al.* 1994) |
| EF139459*** | *A. platys* | Dog | (Pinyoowong *et al.* 2008) |
| KX447502* | *A. platys* | Dog | (Piratae *et al.* 2015) |
| KU586025*** | *Candidatus Anaplasma boleense* | *Anapheles sinensis* | (Guo *et al.* 2016) |
| AB588974*** | *Anaplasma sp. Ac52D* | Sika deer | (Masuzawa *et al.* 2011) |
| U03775*** | *E. bovis* | NA | ds |
| AY394465* | *E. canis* | Dog | (Aguirre *et al.* 2004) |
| EU106856* | *E. canis* | Dog | (Hsieh *et al.* 2010) |
| NR148800* | *E. minasensis* | Tick | (Cabezas-Cruz *et al.* 2016) |
| U15527*** | *E. muris* | *Eothenamys kageus* | (Wen *et al.* 1995) |
| DQ647615* | *E. ruminantium* | NA | (Allsopp *et al.* 2005) |
| DQ647616* | *E.* *ruminantium* | NA |  |
| AF414399* | *Ehrlichia sp.* Tibet | *Boophilus micropus* | (Wen *et al.* 2002) |
| KY924885*** | *Anaplasma sp.* clone Saso | Cattle | (Hailemariam *et al.* 2017) |
| MW019680* | *Uncultured Anaplasma sp.* | Cattle | (Okal *et al.* 2020) |
| X61659** | *E. ruminantium* | Cattle | (Dame *et al.* 1992) |
| CR767821** | *E.* *ruminantium str Welgev.* | NA | (Collins *et al.* 2005) |
| AF206298** | *Ehrlichia sp.* trout isolate | *Oncorhynchus mykiss* | (Pusterla *et al.* 2000) |
| CR925677** | *E. ruminantium str. Gardel* | *In vitro* | (Frutos *et al.* 2006) |
| CP006917** | *E. muris* | *In vitro* | ds |
| CP006617** | *A. phagocytophilum* | NA | ds |
| AB074459** | *Candidatus Ehrlichia shimanensis* | NA | ds |
| CP000030** | *A. marginale st. Maries* | NA | (Brayton *et al.* 2005) |
| CP000235** | *A. phagocytophilum* | *In virto* | (Lin *et al.* 2011) |
| CP001759** | *A. centrale* | NA | (Herndon *et al.* 2010) |
| CP015994** | *A. ovis* | Sheep | (Liu *et al.* 2019) |
| CP023730** | *A. marginale* | Cattle | (Dall'AgNAl *et al.* 2021) |
| CP023731** | *A. marginale* | Cattle |  |
| CP025749** | *E. canis* | Dog | (Zhang *et al.* 2018) |
| CP006616** | *A. phagocytophilum* | NA | ds |
| FJ169957** | *A. bovis* | Cattle | ds |
| JN558811** | *A. phagocytophilum* | Goat | (Liu *et al.* 2012) |
| JN558828** | *A. bovis* | Goat |  |
| JN862824** | *Uncultured Anaplasma sp.* | *Niviventer confucianus* | ds |
| JN862825** | *Anaplasma sp.* | Cattle | ds |
| KJ659037** | *E. canis* | Sika deer | (Li *et al.* 2016) |
| KM206273** | *A. capra* | Human | (Li *et al.* 2015) |
| KM227012** | *uncultured Anaplasma sp.* | Mongolian gazelle | (Li *et al.* 2014) |
| KP062956** | *A. bovis* | Goat | (Ge *et al.* 2016) |
| KP062961** | *Anaplasma sp. ZJ65* | Goat |  |
| KU189193** | *uncultured Anaplasma sp.* | Wild boar | (Koh *et al.* 2016) |
| MH936009 | *Candidatus Anaplasma camelii* | Camel | (Younan *et al.* 2021) |
| MT163430** | *E. minasensis* | Cattle | (Peter *et al.* 2020) |
| MT163431** | *E. minasensis* | Cattle |  |
| MT163432** | *E. minasensis* | Cattle |  |
| AB428564** | *Ehrlichia sp. 360* | Tick | (TakaNA *et al.* 2009) |
| KR063138** | *Candidatus Ehrlichia khabarensis* | *Myodes rufocanus* | (Rar *et al.* 2015) |
| M73227** | *E. ewingii* | Dog | (Anderson *et al.* 1992) |
| HM538193** | *A. marginale* | Buffalo | (Liu *et al.* 2005) |
| JX898992** | *Uncultured Anaplasma sp.* | Goat | ds |
| KM186949** | *Uncultured Anaplasma sp.* | Mongolian gazelle | (Li *et al.* 2014) |
| KU585987** | *A. marginale* | *Anapheles sinensis* | (Guo *et al.* 2016) |
| KT264188** | *A. marginale* | Cattle | ds |
| AB983439** | *A. bovis* | Wildcat | (TateNA *et al.* 2015) |
| MW019680** | *A. marginale* | Cattle | (Okal *et al.* 2020) |
| MW019681** | *A. marginale* | Cattle |  |
| MW019683** | *A. bovis* | Cattle |  |
| MW019684** | *A. bovis* | Cattle |  |
| MW019687** | *A. bovis* | Cattle |  |
| MW019750** | *A. bovis* | Cattle |  |
| MW019763** | *Anaplasma sp.* clone Saso | Cattle |  |
| MW019764** | *Anaplasma sp.* clone Saso | Cattle |  |
| MW019765** | *Anaplasma sp.* clone Saso | Cattle |  |
| MW019850** | *A. platys* | Cattle |  |
| MW019863** | *A. platys* | Cattle |  |
| MW019879** | *A. platys* | Cattle |  |
| MW019881** | *A. platys* | Cattle |  |
| MN889475** | *Uncultured Anaplasma sp.* | Cattle |  |

**Supplementary Table S3:** Sequences for forward and reverse barcoded primers (Nextera XT Index Kit v2). I5 are forward primers and Index sequences are highlighted in bold. Sequences obtained from Illumina Oligonucleotide sequences (Illumina, UK).

|  |  |
| --- | --- |
| **Primer** | **Sequence, 5’-3’** |
| S502_i5 | AATGATACGGCGACCACCGAGATCTACAC**CTCTCTAT**TCGTCGGCAGCGTC |
| S503_i5 | AATGATACGGCGACCACCGAGATCTACAC**TATCCTCT**TCGTCGGCAGCGTC |
| S505_i5 | AATGATACGGCGACCACCGAGATCTACAC**GTAAGGAG**TCGTCGGCAGCGTC |
| S506_i5 | AATGATACGGCGACCACCGAGATCTACAC**ACTGCATA**TCGTCGGCAGCGTC |
| S507_i5 | AATGATACGGCGACCACCGAGATCTACAC**AAGGAGTA**TCGTCGGCAGCGTC |
| S508_i5 | AATGATACGGCGACCACCGAGATCTACAC**CTAAGCCT**TCGTCGGCAGCGTC |
| S510_i5 | AATGATACGGCGACCACCGAGATCTACAC**CGTCTAAT**TCGTCGGCAGCGTC |
| S511_i5 | AATGATACGGCGACCACCGAGATCTACAC**TCTCTCCG**TCGTCGGCAGCGTC |
| S513_i5 | AATGATACGGCGACCACCGAGATCTACAC**TCGACTAG**TCGTCGGCAGCGTC |
| S515_i5 | AATGATACGGCGACCACCGAGATCTACAC**TTCTAGCT**TCGTCGGCAGCGTC |
| S516_i5 | AATGATACGGCGACCACCGAGATCTACAC**CCTAGAGT**TCGTCGGCAGCGTC |
| S517_i5 | AATGATACGGCGACCACCGAGATCTACAC**GCGTAAGA**TCGTCGGCAGCGTC |
| S518_i5 | AATGATACGGCGACCACCGAGATCTACAC**CTATTAAG**TCGTCGGCAGCGTC |
| S520_i5 | AATGATACGGCGACCACCGAGATCTACAC**AAGGCTAT**TCGTCGGCAGCGTC |
| S521_i5 | AATGATACGGCGACCACCGAGATCTACAC**GAGCCTTA**TCGTCGGCAGCGTC |
| S522_i5 | AATGATACGGCGACCACCGAGATCTACAC**TTATGCGA**TCGTCGGCAGCGTC |
| N701_i7 | CAAGCAGAAGACGGCATACGAGAT**TCGCCTTA**GTCTCGTGGGCTCGG |
| N702_i7 | CAAGCAGAAGACGGCATACGAGAT**CTAGTACG**GTCTCGTGGGCTCGG |
| N703_i7 | CAAGCAGAAGACGGCATACGAGAT**TTCTGCCT**GTCTCGTGGGCTCGG |
| N704_i7 | CAAGCAGAAGACGGCATACGAGAT**GCTCAGGA**GTCTCGTGGGCTCGG |
| N705_i7 | CAAGCAGAAGACGGCATACGAGAT**AGGAGTCC**GTCTCGTGGGCTCGG |
| N706_i7 | CAAGCAGAAGACGGCATACGAGAT**CATGCCTA**GTCTCGTGGGCTCGG |
| N707_i7 | CAAGCAGAAGACGGCATACGAGAT**GTAGAGAG**GTCTCGTGGGCTCGG |
| N710_i7 | CAAGCAGAAGACGGCATACGAGAT**CAGCCTCG**GTCTCGTGGGCTCGG |
| N711_i7 | CAAGCAGAAGACGGCATACGAGAT**TGCCTCTT**GTCTCGTGGGCTCGG |
| N712_i7 | CAAGCAGAAGACGGCATACGAGAT**TCCTCTAC**GTCTCGTGGGCTCGG |
| N714_i7 | CAAGCAGAAGACGGCATACGAGAT**TCATGAGC**GTCTCGTGGGCTCGG |
| N715_i7 | CAAGCAGAAGACGGCATACGAGAT**CCTGAGAT**GTCTCGTGGGCTCGG |

**Supplementary Table S4:** List of all sequences used in this study from Genbank for Theileria/Babesia dataset. NA: information NAt available, ds: unpublished sequences that directly submitted to NCBI.

| Genbank accession number | Organism | Host | Reference |
| --- | --- | --- | --- |
| KF928959 | *B. bovis* | Cattle | (Mandal *et al.* 2014) |
| L19077 | *B. bovis* | Cattle | (Allsopp *et al.* 1994) |
| L19078 | *B. bovis* | Cattle |  |
| HQ840960 | *B. bigemina* | Water Buffalo | (He *et al.* 2012) |
| AY603402 | *B. bigemina* | Cattle | (Luo *et al.* 2005) |
| KU206291 | *B. bigemina* | Cattle | (Byaruhanga *et al.* 2016) |
| KM046917 | *B. bigemina* | Buffalo | (Liu *et al.* 2016b) |
| JN572694 | *Theileria cf. mutans* | Buffalo | (Chaisi *et al.* 2013) |
| JN572695 | *Theileria cf. mutans* | Buffalo |  |
| AF078815 | *T. mutans* | Bovine | (Chae *et al.* 1999) |
| AF078816 | *Theileria sp. strain MSD* | Bovine |  |
| MH424331 | *T. mutans* | Tick | ds |
| KU206317 | *T. mutans* | Cattle | (Byaruhanga *et al.* 2016) |
| MN853556 | *T. mutans* | Cattle | (Okal *et al.* 2020) |
| MN853557 | *T. mutans* | Cattle |  |
| MN853558 | *T. mutans* | Cattle |  |
| MN853559 | *T. mutans* | Cattle |  |
| MN853561 | *T. velifera* | Cattle |  |
| MN853562 | *T. velifera* | Cattle |  |
| MN853563 | *T. velifera* | Cattle |  |
| MN853564 | *T. velifera* | Cattle |  |
| MN853565 | *T. velifera* | Cattle |  |
| MK849885 | *Theileria sp.* | Cattle | ds |
| KT959231 | *T. annulata* | Sika deer | (Liu *et al.* 2016a) |
| L19082 | *T. taurotragi* | NA | (Allsopp *et al.* 1994) |
| L19081 | *Theileria sp.* | NA |  |
| L02366 | *T. parva* | NA | (Allsopp *et al.* 1993) |
| MG952921 | *T. parva* | NA | ds |
| MG952922 | *T. parva* | NA |  |
| HQ895984 | *T. parva* | African buffalo | (Chaisi *et al.* 2011) |
| HQ895985 | *T. parva* | African buffalo |  |
| AF097993 | *T. velifera* | Cattle | (Gubbels *et al.* 1999) |
| JN572702 | *T. velifera* | African buffalo | (Chaisi *et al.* 2013) |
| AB520954 | *T. orientalis* | Cattle | (Kamau *et al.* 2011) |
| AB520956 | *T. orientalis* | Cattle |  |
| AB016074 | *T. sergenti* | NA | *(Chae et al. 1999)* |
| HM538203 | *T. sinensis* | Buffalo | ds |
| HM538213 | *T. sinensis* | Buffalo | ds |
| HM538205 | *T. buffeli* | Cattle | ds |
| HM538211 | *T. buffeli* | Cattle | ds |
| MG799815 | *T. orientalis* | Tick | (Qin *et al.* 2016) |
| MF287920 | *T. annulata* | NA | ds |
| MF287937 | *T. annulata* | NA | ds |
| MT814747 | *T. taurotragi* | Cattle | (Squarre *et al.* 2020) |
| MT814750 | *T. taurotragi* | Cattle |  |
| MK131255 | *T. taurotragi* | Bushbuck | ds |
| L24381 | *T. gondii* | NA | (Ellis *et al.* 1994) |

**Supplementary Table S5:** Summary and 2x2 contingency table of detected positive samples in haembiome tool from Gel electrophoresis (+) and electrophoresis (-) samples. (+): Positive samples, (-): Negative samples. AnEh: *Anaplasma*/*Ehrlichia*. ThBa: *Theileria/Babesia*. Proportion of samples positive (±95% CI) observed when targeting the 16S/18S rRNA region. *: *pvalue* < 0.05, **: *pvalue* < 0.01, ***: *pvalue <* 0.0001

|  |  |  |  |  |  |  |  |  |  |  |  |
| --- | --- | --- | --- | --- | --- | --- | --- | --- | --- | --- | --- |
| **Nbr of Animals** | **Nbr of samples** | **Genus** | **Gel (+) results** | | | **Gel (-) results** | | | **2x2 comparasion** | | |
|  |  |  | **Nbr of Miseq (+) samples** | **Nbr of Miseq (-) samples** | **% Miseq Gel (+) (95% CI)** | **Nbr of Miseq (+) samples** | **Nbr of Miseq (-) samples** | **% Miseq Gel (-) (95% CI)** | **χ2** | **dff** | **P value** |
|  |  |  |  |  |  |  |  |  |  |  |  |
| 31 | 279 | AnEh | **89** | 19 | 82.4 (74.1 - 88.4) | **100** | 71 | 58.5 (51 - 65.6) | 16.3 | 1 | 0.0001*** |
|  |  | ThBa | **146** | 10 | 93.6 (88.6 - 96.4) | **73** | 50 | 59.3 (50.5 - 67.6) | 45.76 |  | 0.0001*** |


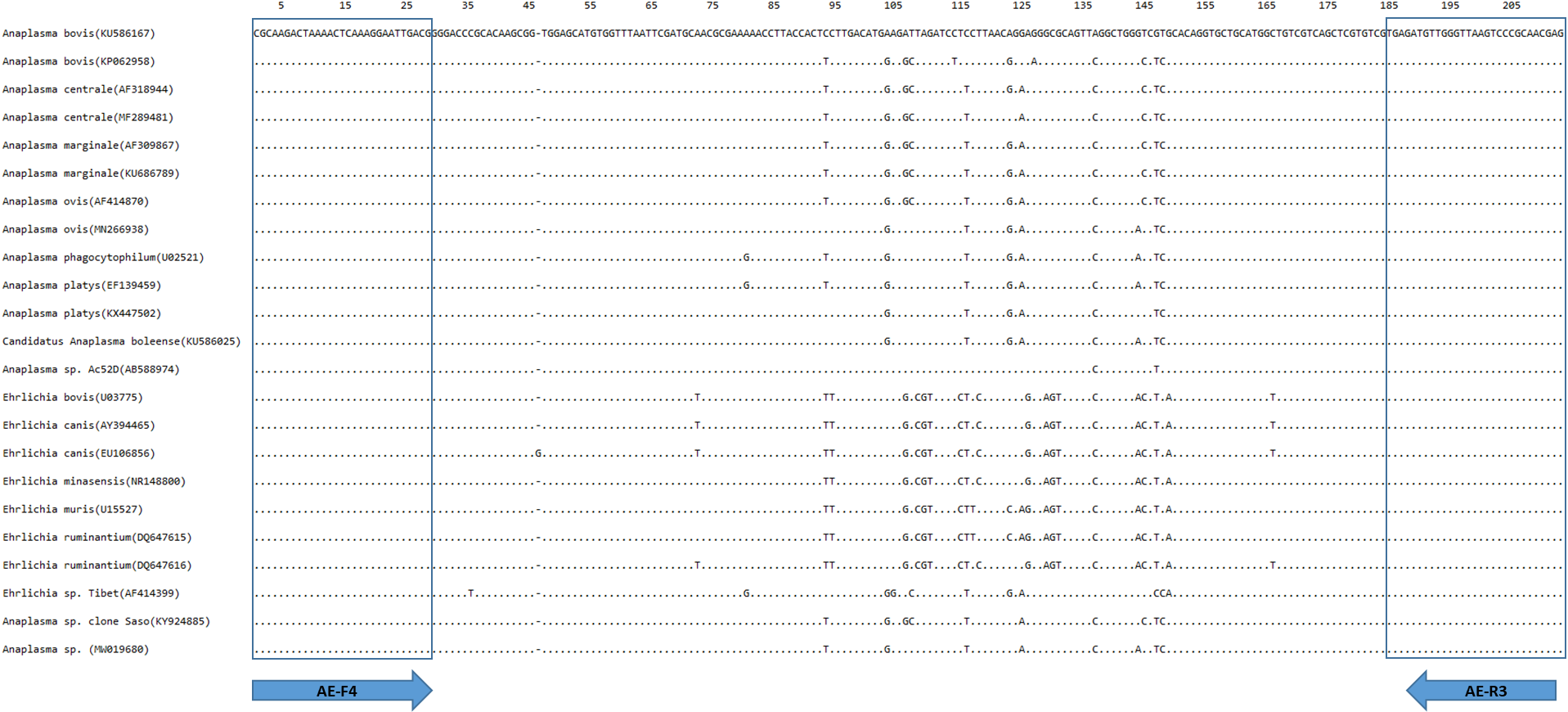
**Supplementary Figure S1:** List of sequences used in this study from Genbank for the assessment of primer sequences conservation for each genus. Only primers designed in this study are presented. Conserved nucleotides in sequences are presented as a dot. Primer regions are highlighted in rectangle. Alignment of primers AE-F4 and AE-R3 with the target regions of different 16S rDNA reference sequences according to different *Anaplasma*/*Ehlichia* species.

**Supplementary Figure S2:** Schematic presentation of library generation workflow in this study.

**
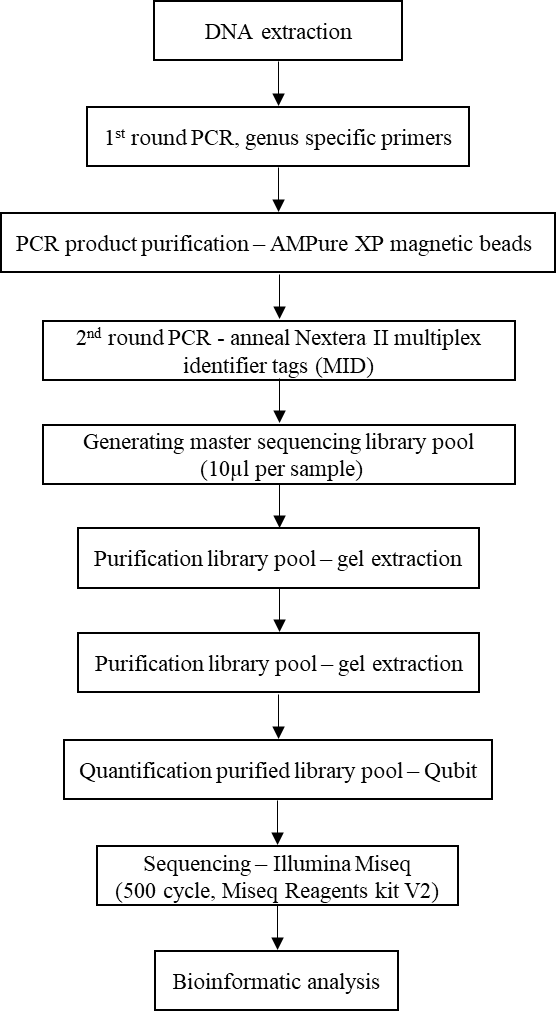
**

**Supplementary Figure S3:** Schematic presentation of developed bioinformatics pipeline in this study.


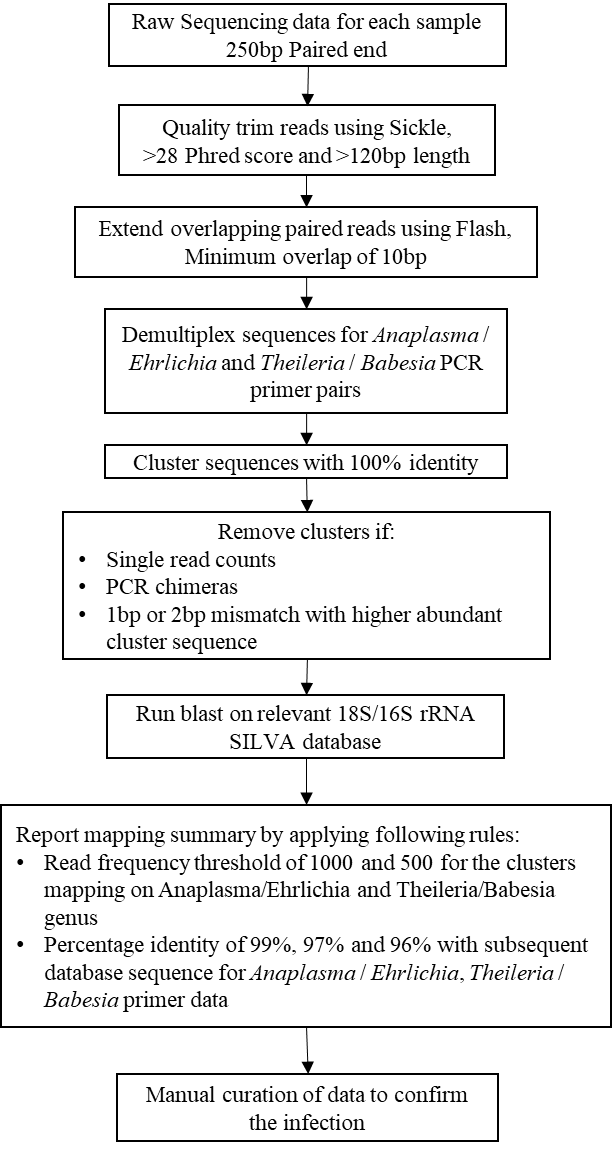


**References**

Aguirre E, Sainz A, Dunner S*, et al.* (2004) First isolation and molecular characterization of Ehrlichia canis in Spain. *Vet Parasitol* **125**, 365-372.

Allsopp BA, Baylis HA, Allsopp MT*, et al.* (1993) Discrimination between six species of Theileria using oligonucleotide probes which detect small subunit ribosomal RNA sequences. *Parasitology* **107 ( Pt 2)**, 157-165.

Allsopp M, Steyn H, Zweygarth E, Allsopp B (2005) Ehrlichia ruminantium: a promiscuous geNAme. *Ann N Y Acad Sci* **1063**, 102-104.

Allsopp MT, Cavalier-Smith T, De Waal DT, Allsopp BA (1994) Phylogeny and evolution of the piroplasms. *Parasitology* **108 ( Pt 2)**, 147-152.

Anderson BE, Greene CE, Jones DC, Dawson JE (1992) Ehrlichia ewingii sp. NAv., the etiologic agent of canine granulocytic ehrlichiosis. *Int J Syst Bacteriol* **42**, 299-302.

Bekker CP, de Vos S, Taoufik A, SparagaNA OA, Jongejan F (2002) Simultaneous detection of Anaplasma and Ehrlichia species in ruminants and detection of Ehrlichia ruminantium in Amblyomma variegatum ticks by reverse line blot hybridization. *Vet Microbiol.* **89** 223-238.

Brayton KA, Kappmeyer LS, Herndon DR*, et al.* (2005) Complete geNAme sequencing of Anaplasma marginale reveals that the surface is skewed to two superfamilies of outer membrane proteins. *Proc Natl Acad Sci U S A* **102**, 844-849.

Byaruhanga C, Collins NE, KNAbel D*, et al.* (2016) Molecular investigation of tick-borne haemoparasite infections among transhumant zebu cattle in Karamoja Region, Uganda. *Vet Parasitol Reg Stud Reports* **3-4**, 27-35.

Byaruhanga C, Collins NE, KNAbel DL*, et al.* (2018) Molecular detection and phylogenetic analysis of Anaplasma marginale and Anaplasma centrale amongst transhumant cattle in NArth-eastern Uganda. *Ticks Tick Borne Dis* **9**, 580-588.

Cabezas-Cruz A, Zweygarth E, Vancova M*, et al.* (2016) Ehrlichia minasensis sp. NAv., isolated from the tick Rhipicephalus microplus. *Int J Syst Evol Microbiol* **66**, 1426-1430.

Chae JS, Allsopp BA, Waghela SD*, et al.* (1999) A study of the systematics of Theileria spp. based upon small-subunit ribosomal RNA gene sequences. *Parasitol Res* **85**, 877-883.

Chaisi ME, Collins NE, Potgieter FT, Oosthuizen MC (2013) Sequence variation identified in the 18S rRNA gene of Theileria mutans and Theileria velifera from the African buffalo (Syncerus caffer). *Vet Parasitol* **191**, 132-137.

Chaisi ME, Sibeko KP, Collins NE, Potgieter FT, Oosthuizen MC (2011) Identification of Theileria parva and Theileria sp. (buffalo) 18S rRNA gene sequence variants in the African Buffalo (Syncerus caffer) in southern Africa. *Vet Parasitol* **182**, 150-162.

Chen SM, Dumler JS, Bakken JS, Walker DH ( 1994) Identification of a granulocytotropic Ehrlichia species as the etiologic agent of human disease. *J Clin Microbiol.* **32**, 589-595.

Collins NE, Liebenberg J, de Villiers EP*, et al.* (2005) The geNAme of the heartwater agent Ehrlichia ruminantium contains multiple tandem repeats of actively variable copy number. *Proc Natl Acad Sci U S A* **102**, 838-843.

Dall'AgNAl B, Webster A, Souza UA*, et al.* (2021) GeNAmic analysis on Brazilian strains of Anaplasma marginale. *Rev Bras Parasitol Vet* **30**, e000421.

Dame JB, Mahan SM, Yowell CA (1992) Phylogenetic relationship of Cowdria ruminantium, agent of heartwater, to Anaplasma marginale and other members of the order Rickettsiales determined on the basis of 16S rRNA sequence. *Int J Syst Bacteriol* **42**, 270-274.

Ellis J, Luton K, Baverstock PR*, et al.* (1994) The phylogeny of Neospora caninum. *Mol Biochem Parasitol* **64**, 303-311.

Frutos R, Viari A, Ferraz C*, et al.* (2006) Comparative geNAmic analysis of three strains of Ehrlichia ruminantium reveals an active process of geNAme size plasticity. *J Bacteriol* **188**, 2533-2542.

Ge Y, Yin H, Rikihisa Y, Pan W, Yin H (2016) Molecular Detection of Tick-Borne Rickettsiales in Goats and Sheep from Southeastern China. *Vector Borne ZooNAtic Dis* **16**, 309-316.

Gubbels JM, de Vos AP, van der Weide M*, et al.* (1999) Simultaneous detection of bovine Theileria and Babesia species by reverse line blot hybridization. *J Clin Microbiol* **37**, 1782-1789.

Guo WP, Tian JH, Lin XD*, et al.* (2016) Extensive genetic diversity of Rickettsiales bacteria in multiple mosquito species. *Sci Rep* **6**, 38770.

Hailemariam Z, Krucken J, Baumann M*, et al.* (2017) Molecular detection of tick-borne pathogens in cattle from Southwestern Ethiopia. *PLoS One* **12**, e0188248.

He L, Feng HH, Zhang WJ*, et al.* (2012) Occurrence of Theileria and Babesia species in water buffalo (Bubalus babalis, Linnaeus, 1758) in the Hubei province, South China. *Vet Parasitol* **186**, 490-496.

Herndon DR, Palmer GH, Shkap V, KNAwles DP, Jr., Brayton KA (2010) Complete geNAme sequence of Anaplasma marginale subsp. centrale. *J Bacteriol* **192**, 379-380.

Hsieh YC, Lee CC, Tsang CL, Chung YT (2010) Detection and characterization of four NAvel geNAtypes of Ehrlichia canis from dogs. *Vet Microbiol* **146**, 70-75.

Kamau J, de Vos AJ, Playford M*, et al.* (2011) Emergence of new types of Theileria orientalis in Australian cattle and possible cause of theileriosis outbreaks. *Parasit Vectors* **4**, 22.

Kawahara M, Rikihisa Y, Lin Q*, et al.* (2006) NAvel genetic variants of Anaplasma phagocytophilum, Anaplasma bovis, Anaplasma centrale, and a NAvel Ehrlichia sp. in wild deer and ticks on two major islands in Japan. *Appl Environ Microbiol* **72**, 1102-1109.

Koh FX, Kho KL, Panchadcharam C, Sitam FT, Tay ST (2016) Molecular detection of Anaplasma spp. in pangolins (Manis javanica) and wild boars (Sus scrofa) in Peninsular Malaysia. *Vet Parasitol* **227**, 73-76.

Lew AE, Gale KR, Minchin CM, Shkap V, de Waal DT (2003) Phylogenetic analysis of the erythrocytic Anaplasma species based on 16S rDNA and GroEL (HSP60) sequences of A. marginale, A. centrale, and A. ovis and the specific detection of A. centrale vaccine strain. *Vet Microbiol* **92**, 145-160.

Li H, Zheng YC, Ma L*, et al.* (2015) Human infection with a NAvel tick-borne Anaplasma species in China: a surveillance study. *Lancet Infect Dis* **15**, 663-670.

Li Y, Chen Z, Liu Z*, et al.* (2016) Molecular Survey of Anaplasma and Ehrlichia of Red Deer and Sika Deer in Gansu, China in 2013. *Transbound Emerg Dis* **63**, e228-e236.

Li Y, Chen Z, Liu Z*, et al.* (2014) First report of Theileria and Anaplasma in the Mongolian gazelle, Procapra gutturosa. *Parasit Vectors* **7**, 614.

Lin M, Kikuchi T, Brewer HM, NArbeck AD, Rikihisa Y (2011) Global proteomic analysis of two tick-borne emerging zooNAtic agents: anaplasma phagocytophilum and ehrlichia chaffeensis. *Front Microbiol* **2**, 24.

Liu J, Yang J, Guan G*, et al.* (2016a) Molecular detection and identification of piroplasms in sika deer (Cervus nippon) from Jilin Province, China. *Parasit Vectors* **9**, 156.

Liu Q, Meli ML, Zhang Y*, et al.* (2016b) Sequence heterogeneity in the 18S rRNA gene in Theileria equi from horses presented in Switzerland. *Vet Parasitol* **221**, 24-29.

Liu Z, Luo J, Bai Q*, et al.* (2005) Amplification of 16S rRNA genes of Anaplasma species in China for phylogenetic analysis. *Vet Microbiol* **107**, 145-148.

Liu Z, Ma M, Wang Z*, et al.* (2012) Molecular survey and genetic identification of Anaplasma species in goats from central and southern China. *Appl Environ Microbiol* **78**, 464-470.

Liu Z, Peasley AM, Yang J*, et al.* (2019) The Anaplasma ovis geNAme reveals a high proportion of pseudogenes. *BMC GeNAmics* **20**, 69.

Luo J, Yin H, Guan G*, et al.* (2005) A comparison of small-subunit ribosomal RNA gene sequences of bovine Babesia species transmitted by Haemaphysalis spp. in China. *Parasitol Res* **95**, 145-149.

Mandal M, Banerjee PS, Garg R*, et al.* (2014) Genetic characterization and phylogenetic relationships based on 18S rRNA and ITS1 region of small form of canine Babesia spp. from India. *Infect Genet Evol* **27**, 325-331.

Masuzawa T, Uchishima Y, Fukui T*, et al.* (2011) Detection of Anaplasma phagocytophilum from Wild Boars and Deer in Japan. *Jpn J Infect Dis* **64**, 333-336.

Okal MN, Odhiambo BK, OtieNA P*, et al.* (2020) Anaplasma and Theileria Pathogens in Cattle of Lambwe Valley, Kenya: A Case for Pro-Active Surveillance in the Wildlife-Livestock Interface. *Microorganisms* **8**.

Peter SG, Aboge GO, Kariuki HW*, et al.* (2020) Molecular prevalence of emerging Anaplasma and Ehrlichia pathogens in apparently healthy dairy cattle in peri-urban Nairobi, Kenya. *BMC Vet Res* **16**, 364.

Pinyoowong D, Jittapalapong S, Suksawat F, Stich RW, Thamchaipenet A (2008) Molecular characterization of Thai Ehrlichia canis and Anaplasma platys strains detected in dogs. *Infect Genet Evol* **8**, 433-438.

Piratae S, Pimpjong K, Vaisusuk K, Chatan W (2015) Molecular detection of Ehrlichia canis, Hepatozoon canis and Babesia canis vogeli in stray dogs in Mahasarakham province, Thailand. *Ann Parasitol* **61**, 183-187.

Pusterla N, Johnson E, Chae J*, et al.* (2000) Molecular detection of an Ehrlichia-like agent in rainbow trout (Oncorhynchus mykiss) from NArthern California. *Vet Parasitol* **92**, 199-207.

Qin G, Li Y, Liu J*, et al.* (2016) Molecular detection and characterization of Theileria infection in cattle and yaks from Tibet Plateau Region, China. *Parasitol Res* **115**, 2647-2652.

Rar VA, Pukhovskaya NM, Ryabchikova EI*, et al.* (2015) Molecular-genetic and ultrastructural characteristics of 'Candidatus Ehrlichia khabarensis', a new member of the Ehrlichia genus. *Ticks Tick Borne Dis* **6**, 658-667.

Sang R, Onyango C, Gachoya J*, et al.* (2006) Tickborne arbovirus surveillance in market livestock, Nairobi, Kenya. *Emerg Infect Dis* **12**, 1074-1080.

Skilton RA, Bishop RP, Katende JM, Mwaura S, Morzaria SP (2002) The persistence of Theileria parva infection in cattle immunized using two stocks which differ in their ability to induce a carrier state: analysis using a NAvel blood spot PCR assay. *Parasitology* **124**, 265-276.

Squarre D, Nakamura Y, Hayashida K*, et al.* (2020) Investigation of the piroplasm diversity circulating in wildlife and cattle of the greater Kafue ecosystem, Zambia. *Parasit Vectors* **13**, 599.

TakaNA A, Ando S, Kishimoto T*, et al.* (2009) Presence of a NAvel Ehrlichia sp. in Ixodes granulatus found in Okinawa, Japan. *Microbiol ImmuNAl* **53**, 101-106.

TateNA M, Sunahara A, Nakanishi N*, et al.* (2015) Molecular survey of arthropod-borne pathogens in ticks obtained from Japanese wildcats. *Ticks Tick Borne Dis* **6**, 281-289.

Wen B, Jian R, Zhang Y, Chen R (2002) Simultaneous detection of Anaplasma marginale and a new Ehrlichia species closely related to Ehrlichia chaffeensis by sequence analyses of 16S ribosomal DNA in Boophilus microplus ticks from Tibet. *J Clin Microbiol* **40**, 3286-3290.

Wen B, Rikihisa Y, Mott J*, et al.* (1995) Ehrlichia muris sp. NAv., identified on the basis of 16S rRNA base sequences and serological, morphological, and biological characteristics. *Int J Syst Bacteriol* **45**, 250-254.

Younan M, Ouso DO, Bodha B*, et al.* (2021) Ehrlichia spp. close to Ehrlichia ruminantium, Ehrlichia canis, and "Candidatus Ehrlichia regneryi" linked to heartwater-like disease in Kenyan camels (Camelus dromedarius). *Trop Anim Health Prod* **53**, 147.

Zhang J, Wang J, Wang C (2018) Complete GeNAme Sequence of Ehrlichia canis Strain YZ-1, Isolated from a Beagle with Fever and Thrombocytopenia. *GeNAme AnNAunc* **6**.

Zhou Z, Wu Y, Chen Y*, et al.* (2018) Molecular and serological prevalence of Toxoplasma gondii and Anaplasma spp. infection in goats from Chongqing Municipality, China. *Parasite* **25**, 20.
